# PAGER Web APP: An Interactive, Online Gene Set and Network Interpretation Tool for Functional Genomics

**DOI:** 10.3389/fgene.2022.820361

**Published:** 2022-04-12

**Authors:** Zongliang Yue, Radomir Slominski, Samuel Bharti, Jake Y. Chen

**Affiliations:** ^1^ Informatics Institute in the School of Medicine, The University of Alabama at Birmingham, Birmingham, AL, United States; ^2^ Graduate Biomedical Sciences Program, The University of Alabama at Birmingham, Birmingham, AL, United States

**Keywords:** PAGER, melanoma, functional genomics, geneset analysis, network visualization and analysis, PAGER Web APP, GNPA

## Abstract

Functional genomics studies have helped researchers annotate differentially expressed gene lists, extract gene expression signatures, and identify biological pathways from omics profiling experiments conducted on biological samples. The current geneset, network, and pathway analysis (GNPA) web servers, e.g., DAVID, EnrichR, WebGestaltR, or PAGER, do not allow automated integrative functional genomic downstream analysis. In this study, we developed a new web-based interactive application, “PAGER Web APP”, which supports online R scripting of integrative GNPA. In a case study of melanoma drug resistance, we showed that the new PAGER Web APP enabled us to discover highly relevant pathways and network modules, leading to novel biological insights. We also compared PAGER Web APP’s pathway analysis results retrieved among PAGER, EnrichR, and WebGestaltR to show its advantages in integrative GNPA. The interactive online web APP is publicly accessible from the link, https://aimed-lab.shinyapps.io/PAGERwebapp/.

## Introduction

Functional genomics analysis is widely performed to characterize genes and intergenic regulatory regions in the genome that contribute to different biological processes (Yang et al., 2020; Angeloni et al., 2021). Essentially, functional genomics provides a way to reveal the molecules’ coordination in mechanisms due to a specific phenotype (Raamsdonk et al., 2001; Rahaman et al., 2015). By tracking the molecular activities in the specific biological conditions, we could identify those driver and passenger genes working in a model linking genotype to phenotype. Numerous studies have shown that the molecules working in pathways could help in disease diagnosis (Zhang and Chen, 2010; Drier et al., 2013; Livshits et al., 2015; Bock and Ortea, 2020; Pian et al., 2021), cancer subtyping (Zhang and Chen, 2013; Mallavarapu et al., 2020; Lafferty et al., 2021), and personalized medicine (Chen et al., 2007; Hamburg and Collins, 2010; Raghavan et al., 2017). Additionally, multi-omics analysis provides a complex map linking transcriptomics, proteomics, and metabolomics (Subramanian et al., 2020; Andrieux and Chakraborty, 2021). In multi-omics studies, the challenges for functional genomics are the coverage of contents, the rendering of the complex network-based models, and the easy-to-use software with advanced features. Therefore integrative geneset, network, and pathway analysis (GNPA) have emerged in the past decade to lessen the burden of multi-omics data analysis users (Wu et al., 2014). Pathway analysis, especially topology-based approaches that exploit all the knowledge about how genes and proteins interact in a pathway, have been developed to discover the mechanical changes through pathway-level scoring and pathway significance assessment (Draghici et al., 2007; Mitrea et al., 2013; Nguyen et al., 2018). To better understand the impact of perturbations or genetic modifications in a system-level, System-level PAThway Impact AnaLysis using map (SPATIAL), Signaling Pathway Impact Analysis - Global Perturbation Factor (SPIA-GPF), and SPATIAL-GPF have been introduced (Bokanizad et al., 2016).

During the last decade, several GNPA web servers have been developed (Subramanian et al., 2005; Khatri et al., 2012), including DAVID (Jiao et al., 2012), EnrichR (Kuleshov et al., 2016), WebGestalt (Liao et al., 2019), and pathways, annotated gene lists and gene signatures electronic repository (PAGER) (Yue et al., 2018). The highlights of those webservers are interactive and comprehensive data coverage. The first version of the DAVID tool was published in 2003 (Dennis et al., 2003), and it is one of the earliest geneset enrichment analysis webservers. The most updated version of DAVID implements many advanced features such as gene ranking, which gives a quick focus on the most likely important candidate genes, gene with annotation in each single view, and gene extension to make functional inferences (Jiao et al., 2012). EnrichR was initially developed in 2013, and its merits come from comprehensive data coverage and interactive visualization panel (Chen et al., 2013). EnichR provides 190 libraries and adds Appyter to visualize EnrichR results in different styles (Kuleshov et al., 2016). WebGestalt was introduced in 2005 (Zhang et al., 2005), and it highlights the visualization of gene ontology hierarchy structure and pathway view of wikiPathway. WebGestaltR implemented with R language in the recent updates (Liao et al., 2019).

PAGER was initially conceived in 2014 (Harini et al., 2008) and subsequently developed in 2015 (Yue et al., 2015) with a standardized concept called “PAGs” (Pathways, Annotated gene lists, and Gene signatures) that integrates different levels of gene-sets. PAGER highlights the measurement of biological relevance using normalized Cohesion Coefficient (nCoCo) and advances the network interpretation of functional genomics results in several aspects. Additionally, PAGER introduced the computational strategies in generating m-type (co-membership) or r-type (regulatory) PAG-to-PAG relationships. PAGER also provides gene prioritization in each PAG. For the intra-PAG network construction, PAGER adopts the protein-protein interactions from the HAPPI database (Chen et al., 2017), a comprehensive and high-quality map of Human annotated and predicted protein interactions, and gene regulations validated *in vitro* experiment. Hence, PAGER enables gene prioritization using the network topology in each PAG (Yue et al., 2018). All four web servers support API (Application Programming Interface) services.

In this study, we developed the PAGER Web APP, an interactive online application to perform the gene set enrichment analysis and network interpretation of the functional genomics result. PAGER Web APP provides preprocessed RNA-seq data from UALCAN-processed TCGA data (Chandrashekar et al., 2017) and a melanoma drug resistant-sensitive case study (Snyder et al., 2014) from cBioPortal (Gao et al., 2013). We illustrated how the PAGER Web APP enhances the potential to discover biological insights using network-based computational strategy by comparing the enriched pathways from the three leading web servers using their application programming interfaces (APIs). We performed three additional case studies, multiple sclerosis (MS), colonic mucosa in Crohn’s disease (CD), and ulcerative colitis (UC) study, to compare the three web server performances and further validate the pathways using PubMed co-citations. We intend for PAGER Web APP to become a popular application for researchers interested in integrative GNPA.

## Methods

### Workflow and User Interface

**FIGURE 1:**
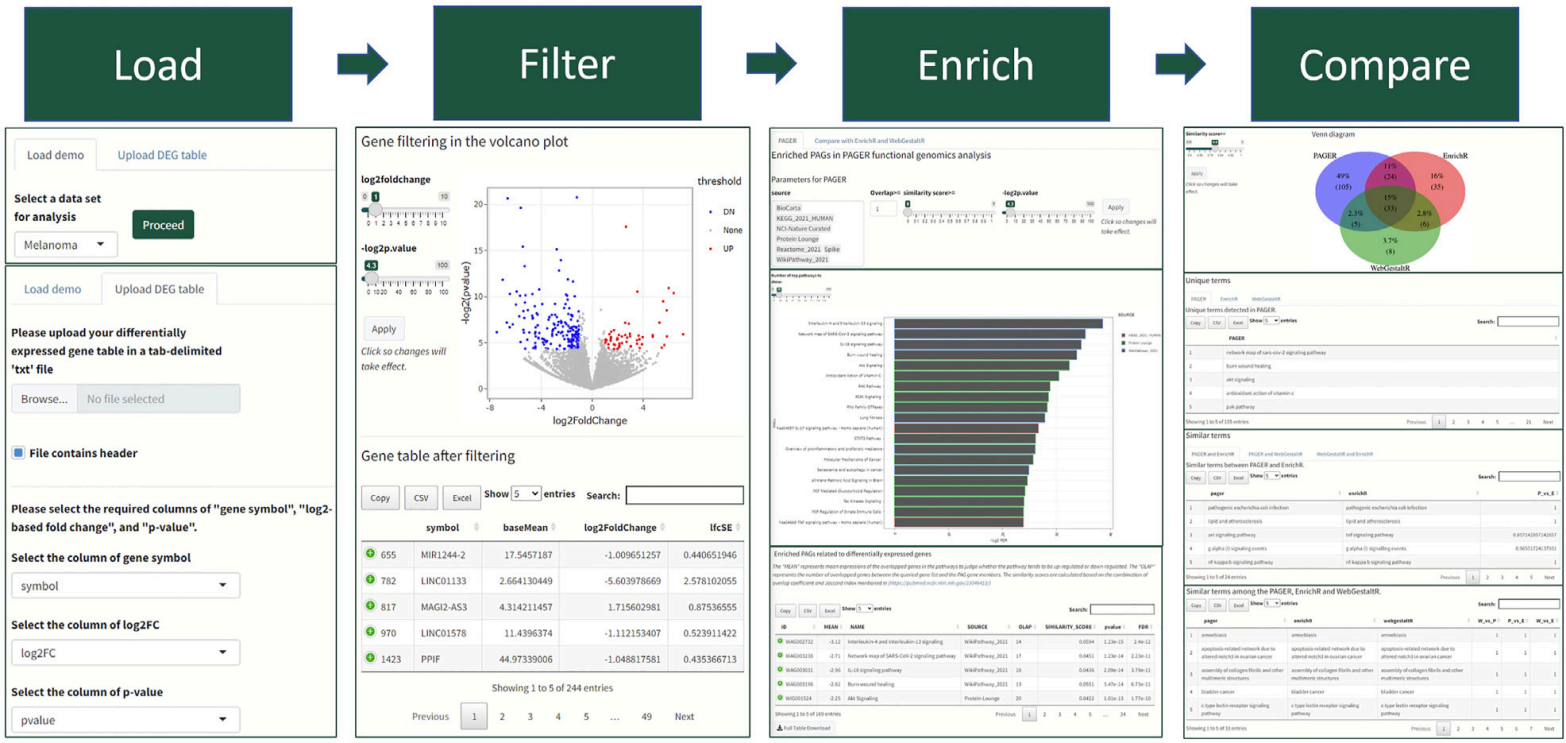
The PAGER Web APP data analysis workflow. The workflow consists of four steps with visualization panels to help biologists quickly understand the results.

We developed a four-step procedure in performing the functional genomics analysis in PAGER Web APP for Human genomics results (Figure 1). Firstly, users need to either load Demo data or upload their data. In the Demo data, PAGER Web APP provides a melanoma dataset, a multiple sclerosis dataset, a Crohn’s disease dataset, an ulcerative colitis dataset and 16 cancer types collected from UALCAN TCGA data (Chandrashekar et al., 2017). If users need to upload the data, we ask users to provide a tab-delimited.txt format file, check the log2 fold change column and *p*-value column, and click on the “proceed” button. Secondly, PAGER Web APP will generate a volcano plot using the gene’s log2 fold changes and colors the over-expressed candidate genes red and under-expressed candidate genes blue using the default threshold *p*-value ≤ 0.05 and absolute log2 fold change ≥1. PAGER Web APP allows users to adjust the log2foldchange and negative log2-based *p*-value to optimize the candidate gene list. Users need to click on the proceed button to the next step. Thirdly, PAGER Web APP will perform the gene-set enrichment analysis with the pathway type geneset sources (P-type PAGs) in default. Users can add or remove the source name in the source multiple-choice field. PAGER Web APP also allows users to change the minimum number of overlapped genes, similarity score, and “-log2*p*-value” cutoff. The similarity score is based on the combination of overlap coefficient and Jaccard index using the methods described previously (Huang et al., 2012). In the table of enriched genesets results, users can use the column “PAGER link” to navigate to the web-hosted PAGER entries of the given PAG, including the metadata, gene members, and gene networks. PAGER Web APP offers two additional leading gene set enrichment analysis tools (EnrichR and WebGestaltR) using the API service. We didn’t include DAVID due to the API failure. Lastly, PAGER Web APP summarizes the similarity of the terms and displays a Venn diagram of the overlapped terms. PAGER Web APP also provides the corresponding tables to deliver similar terms with similarity scores by comparing the three tools. All the tables and plots are downloadable.

### Term to Term Distance-Based Similarity of Terms Enriched From the Three Tools

The term similarity is generated based on a string metric using the Stringdist library (https://cran.r-project.org/web/packages/stringdist/index.html). We clean up the terms or names by removing irrelevant content, such as species, identifier, etc., and making all the terms lower case. We also remove the redundant terms enriched from different data sources, such as “MAPK signaling pathway” may come from KEGG and wikiPathway at the same time. Then we apply the string similarity using optimal string alignment (OSA) distance (Boytsov, 2011) to generate the similarity matrix between two sets of terms, set A and set B.

Assume there are two terms regarded as two strings a and b , the restricted distance is defined as $d_{a,b}\left(i,j\right)$ in a recursive calculation, the i is the prefix of string a , and the j is the prefix of string b .

$$d_{a,b}\left(i,j\right)=\min\{\begin{array}{c} 0\;\;if\;i=j=0\\ d_{a,b}\left(i-1,j\right)+1\;if\;i>0\left(deletion\right)\\ d_{a,b}\left(i,j-1\right)+1\;if\;j>0\left(insertion\right)\\ d_{a,b}\left(i-1,j-1\right)\;if\;a_{i}=b_{j}\left(match\right)\\ d_{a,b}\left(i-1,j-1\right)+1\;if\;a_{i}\neq b_{j}\left(\mathrm{substitution}\right)\\ d_{a,b}\left(i-2,j-2\right)+1\;if\;i,j>1\;\mathrm{and}\;a_{i}=b_{j-1}\;and\;a_{i-1}=b_{j}\left(\mathrm{transposition}\right) \end{array}$$

The string similarity is calculated by:

$$1-\frac{d_{a,b}\left(i,j\right)}{\max\left(\left|a\right|,\left|b\right|\right)}$$

where |a| represents the length of string a , and |b| represents the length of string b .

After generating the similarity matrix between the two lists of terms, we check each row (a term from the set A) and take the highest score with the term as the most similar term. Therefore, we generate a list of pairwise term-to-term similarities. Finally, we use the default or customized similarity cutoff to filter low similar term-to-term pairs.

### Apply Louvain Clustering in m-Type PAG-To-PAG Networks to Identify PAG Communities

We apply the Louvain clustering function in the igraph library in R (https://cran.r-project.org/web/packages/igraph/index.html) to find the community structure in m-type PAG-to-PAG networks. The Louvain clustering is based on the modularity in a scale between -0.5 (non-modular clustering) to 1 (fully modular clustering) described in the paper (Blondel et al., 2008).

### Extract the Critical Concepts From Pathways and Show Them in Word-Clouds

We create bag-of-words from the space-separated PAG names to present the frequently appearing words in each PAG for any enriched PAG set. We create word corpus, remove the potential punctuation such as comma, colon, etc., make all the words lower case, remove both irrelevant words and common words, “pathway,” “signaling,” “human,” “homo,” “sapiens,” “has,” “or,” and “and”. Finally, we apply wordcloud2 function in the wordcloud2 library in R (https://cran.r-project.org/web/packages/wordcloud2/index.html) for the visualization.

### Implementing the Software

The PAGER Web Application user interface is designed using bs4Dash (https://cran.r-project.org/web/packages/bs4Dash/index.html) package in R. The application is supported by R Shiny (https://shiny.rstudio.com/) framework. In addition to data processing and statistical analysis, GNPA Analysis are implemented using PAGER API, EnrichR API and WebGestaltR API. Graphing libraries like Plotly (https://plotly.com/r/), igraph in R (https://igraph.org/r/), ggplot2, wordcloud2, and VennDiagram have been used.

### Prepare the Melanoma Drug Resistant-Sensitive Data From cBioPortal

We downloaded the melanoma dataset of 64 patient samples from cBioPortal, and it was initially published in a paper in the New England Journal of Medicine (Snyder et al., 2014). We identified a cohort from the patients who are in the metastasis stage (m1c) with Neuroblastoma RAS Viral Oncogene Homolog (NRAS gene) or/and v-Raf murine sarcoma viral oncogene homolog B (BRAF gene) mutations. Hence, we obtained three drug response patients, two drug weakly response patients and seven non-response patients. We applied the DEseq2 library in R (https://bioconductor.org/packages/release/bioc/html/DESeq2.html) to generate the differentially expressed genes that compared drug-resistant patients to drug-sensitive patients. The output file is stored in the PAGER Web APP as a demo.

### Prepare the Multiple Sclerosis Data From the EMBL-EBI Database

We loaded the differentially expressed gene table, “extdata/E-GEOD-21942.topTable.RData”, preprocessed in the ROntoTools library (Ansari et al., 2016). This dataset contains a genome-wide array expression study in peripheral blood mononuclear cells (PBMC) from 12 multiple sclerosis (MS) patients and 15 controls (Kemppinen et al., 2011). We selected differentially expressed genes using adjusted *p*-value ≤ 0.01 (2,864 genes) and saved their fold changes as input of ROntoTools. We set the adjusted *p*-value ≤ 0.01 and the absolute logFC >0.5 to get the 1,470 candidate genes as the input of PAGER, EnrichR and WebGestaltR.

### Prepare the Colonic Mucosa Data From the EMBL-EBI Database

We downloaded the transcription profiling by array of RNA from inflamed and non-inflamed colonic mucosa (E-MTAB-2967). In Crohn’s disease, there are 15 inflamed colonic mucosa and 15 controls. In ulcerative colitis, there are 14 inflamed colonic mucosa and 14 controls. We performed the normalization and linear regression using the limma library in R (https://bioconductor.org/packages/release/bioc/html/limma.html). We set the cutoffs of adjusted *p*-value ≤ 0.05 and the absolute logFC >0.5 to get the 518 candidate genes in Crohn’s disease and the 528 candidate genes in the ulcerative colitis study.

### Validation of Pathways Using the Co-citations in PubMed Literature

To demonstrate the significance of the keywords in pathways related to a disease, we applied a co-citation enrichment analysis using the hypergeometric test and odds ratio. We applied the NCBI e-utils application programming interface (API) that implements semantic searches of PubMed abstracts to report biomedical literature citations (Sayers, 2008). We implied that the likelihood of observing articles co-mentioning disease names and the keywords from pathways is statistically higher than random using the PubMed score (Yue et al., 2019a). In this study, the background citations using the word “disease” denoted as N , the citations of the specific disease using the word “melanoma” represented as K , the citations of the keywords from a pathway denoted as n , and the joint citations of “melanoma” and the keywords from a pathway represented as k . We performed the co-citation enrichment analysis to generate PubMed score using the formula:

$$PubMed\;score=-\ln\left(\sum_{t=k}^{\min\left(n,K\right)}\frac{\left(\begin{array}{c} K\\ t \end{array}\right)\left(\begin{array}{c} N-K\\ n-t \end{array}\right)}{\left(\begin{array}{c} N\\ n \end{array}\right)}\right)$$

We calculated the odds ratio based on the formula $\frac{k/\left(K-k\right)}{\left(n-k\right)/\left(N-K-n+k\right)}$ . We also manually checked the contents and subsequently confirmed them using the PubTator annotation API (Wei et al., 2019; Wei et al., 2013), i.e., https://www.ncbi.nlm.nih.gov/research/pubtator-api/publications/export/pubtator?pmids=[PMID]. We took a sample list of PubMed IDs from each retrieved entry. To remove biases and further confirm the mentioned keywords, we applied the analysis described in the previously developed tool called biomedical entity expansion ranking and exploration (BEERE) (Yue et al., 2019b) to extract those semantic relationships that co-mention “melanoma” and the pathways’ keywords.

To evaluate how well the method can identify “correct” pathways, we introduced a new hybrid validation technique. It involves first defining the ground truth and subsequently developing a statistical model to assess the significance of results retrieved using a receiver operating characteristics (ROC) curve and the area-under-the-curve (AUC) value. The hybrid technique also includes performing a literature co-citation-based assessment. We constructed the ground truth using ROntoTools, the best performing method reported in the review paper (Nguyen et al., 2019), in three steps. Firstly, we took the candidate genes from the differential expression analysis using the adjusted *p*-value cutoff 0.05 and the absolute gene’s log fold-changes larger than or equal to 0.5. Secondly, we performed pathway enrichment analysis using the ROntoTools. Thirdly, we defined the “true” data set as the significantly enriched pathways with adjusted combined *p*-values ≤ 0.05 (combined *p*-values were generated by the function “comb.pv.func” (Kemppinen et al., 2011) in ROntoTools) and the “false” data set as the retrieved pathways with adjusted combined *p*-values > 0.05 but with at least one gene overlapping with the input gene list.

In the literature co-citation validation, we developed a t-test based statistical model on evaluating how significant the *p*-value ranked pathways can be supported by the PubMed scores. Particularly, we ranked the PAGs based on adjusted *p*-values, and compared the top n% PAGs’ PubMed scores to the bottom (100-*n*)% PAGs’ PubMed scores for each method, where n ranges from 10 to 90 with a step increment of 10. And then, we reported their average *p*-values, respectively. The smaller *p*-values are, the better performance the methods have.

## Results

### Comparison of Data Coverage and Features Among the Three Web Servers

Compared to EnrichR and WebGestalt, PAGER progresses the network interpretation of functional genomics results. Although there are 35 unique geneset libraries reported in most updated PAGER, which are less than EnrichR, each of PAG in PAGER contains metadata other than EnrichR and WebGestalt, including PAG-type (pathways, annotated gene lists and gene signatures), PAG descriptions, source link, publication reference, curator, and nCoCo score (described in PAGER 2.0). In addition, PAGER provides geneset intra-network views, including the protein-protein interaction network and gene-gene regulation network members in each geneset, while WebGestalt reports pathway maps in wikiPathway source only. For the geneset’s inter-network, WebGestalt inherits the Gene Ontology (GO) hieratical structure from the GO consortium. We extent the relationship concepts by introducing m-type (co-membership) PAG-to-PAG relationships and r-type (regulatory) PAG-to-PAG relationships described in PAGER. The m-type PAG-to-PAG relationships represent co-memberships between two PAGs, which reveals signaling cross-talk between PAGs that share signaling components within signal transduction pathways in response to external stimuli. The r-type PAG-to-PAG relationships represent the PAG causal ordering inferred from gene-to-gene regulations by adapting our method previously described in PAGER (Yue et al., 2018). The PAGER Web APP fulfills all the additional features in Table 1, such as term searching and API service.

**TABLE 1:** A comparison of data coverage and features among PAGER, EnrichR, and WebGestalt web servers.

Webserver		PAGER	EnrichR	Webgestalt
Data coverage (Human)	Unique library	35	89	22
	Metadata	Yes	Partial	Partial
	Gene prioritization	Yes	No	No
Geneset intra-network	Interactions	Yes	No	Partial
	Regulations	Yes	No	Partial
Geneset inter-network	m-type (co-membership)	Yes	Partial	Partial
	r-type (regulatory)	Yes	No	No
Additional feature	Term searching	Yes	Yes	No
	API	Yes	Yes	Yes

### Melanoma Drug Resistant-Sensitive Patients Enriched Pathway Case Study in Demo

To better identify the cohorts in melanoma cancer to improve the treatment, functional genomics has been applied to the next-generation sequencing data for an in-depth understanding of the molecular mechanisms in the drug resistance cases. We collected the transcriptomes from the cBioPortal database in this study. In the result, we found 164 P-type PAGs (pathways) to be significantly enriched.

**FIGURE 2:**
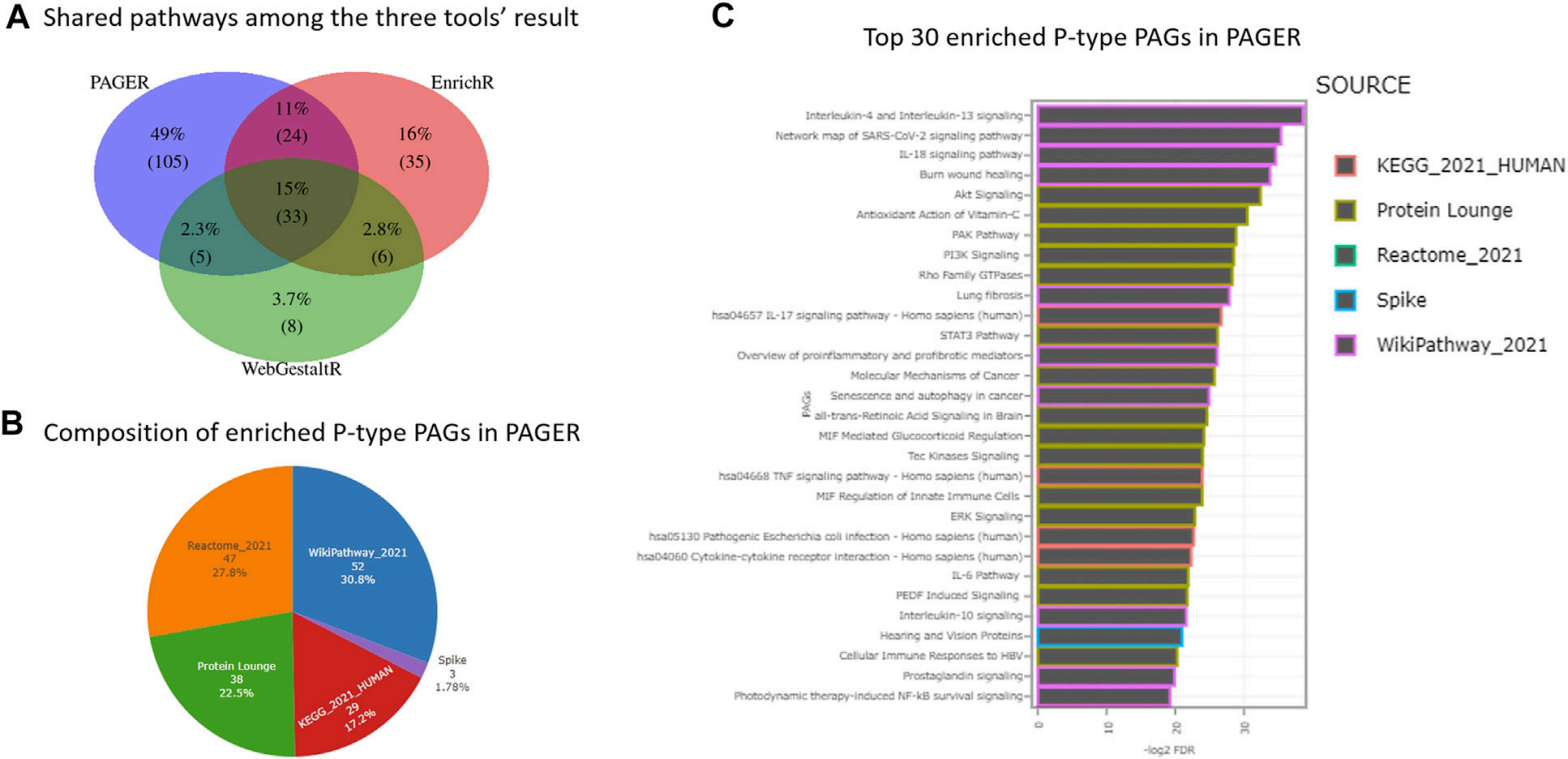
The enriched pathway results for melanoma drug resistant-sensitive patients. **(A)** The consensus pathways among PAGER, EnrichR and WebGestaltR results. **(B)** The composition of P-type PAGs enriched in PAGER. **(C)** The top-30 enriched P-type PAGs are ordered by FDR in PAGER.

In the 164 P-type PAGs, they are two PAGs that are derived from more than one data source, i.e., “PI3K-Akt signaling pathway” and “Bladder cancer”, each of which is simultaneously recorded in both “WikiPathway_2021” and “KEGG_2021_HUMAN” data sources. Compared to the results from EnrichR and WebGestaltR, PAGER had the greatest number of enriched pathways, which is 164, EnrichR has 98, and WebGestaltR has 52 (Figure 2A). PAGER also had the greatest number of unique pathways, which is 101 (48%). We found 33 (16%) overlapped pathways among the three tools. In addition, 23 (11%) pathways were shared between PAGER and EnrichR, 5 (2.4%) pathways were shared between PAGER and WebGestaltR, and 6 (2.8%) pathways were shared between EnrichR and WebgestaltR.

In the 164 P-type PAGs reported by PAGER, there were 4 major sources and 1 minor source (Figure 2B). 50 (30.5%) are from wikiPathway, 46 (28%) are from Reactome, 37 (22.6%) are from Protein Lounge, 28 (17.1%) are from KEGG, and 3 (1.83%) are from Spike. We showed the top-30 enriched P-type PAGs colored by the sources in the horizontal bar-plot in Figure 2C, and the details of the enriched PAGs are in Supplementary Table S1.

### Critical Terms Extraction From the Louvain Clustered PAGs in the m-Type PAG-To-PAG Networks

**FIGURE 3:**
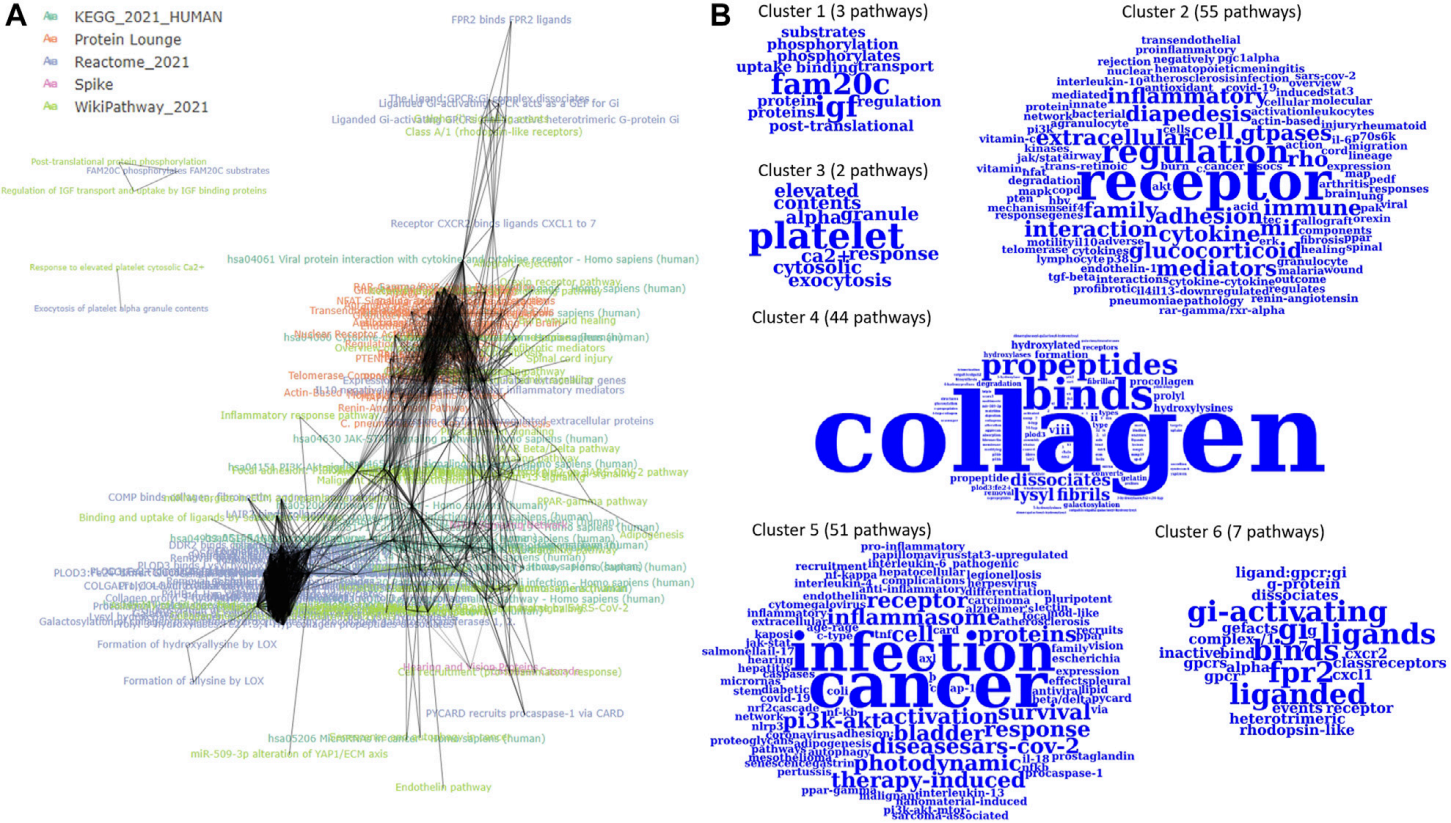
The m-type PAG-to-PAG network of enriched P-type PAGs in PAGER for melanoma drug resistant-sensitive patients. **(A)** The m-type PAG-to-PAG network overview and **(B)** The extracted word clouds from the Louvain clusters in the network.

The 164 P-type PAGs form a densely connected m-type PAG-to-PAG network (2,749 m-type PAG-to-PAG relationships) with an average degree of 18. After the community detection using Louvain clustering, we found 5 PAG clusters in the m-type PAG-to-PAG network (Figure 3). The extracted concepts reveal the general pathway functions in the clusters. **Cluster 1** consists of 3 pathways with represented terms “FAM20C” protein (Golgi-associated secretory pathway kinase), “IGF” protein (insulin-like growth factor). **Cluster 2** has 7 pathways related to the Gi-activating and ligand-receptor bindings. **Cluster 3** is formed by 43 pathways related to collagen formation and binding events. **Cluster 4** has 37 pathways related to inflammasome responses in cancer or infection. **Cluster 5** contains 66 pathways with the regulation of several inflammatory and cytokine responses through the receptor interactions. Hence, PAGER Web APP enables screening for the critical terms and quickly identifying the specific molecular mechanism communities in the m-type PAG-to-PAG network.

### Validation of the Enriched Pathways Using Literature Support in the Melanoma Drug Resistant-Sensitive Study

We found all of them relevant to melanoma cancer for the 33 consensus pathways among PAGER, EnrichR, and WebGestaltR results. We listed the results using pathway name, the pairwise term similarities, keywords used for co-citation retrieval, number of co-citations, odds ratio, PubMed score, one of PubMed IDs, and BEERE validation (in Table 2; Supplementary Table S2). All the pathways were determined to be related to Melanoma with PubMed literature support. For the 23 consensus pathways between PAGER and EnrichR results, we found that all of them have at least one literature support (Table 3). We showed all the BEERE-identified semantic relationships in Supplementary Table S3. We found that all the 5 consensus pathways between PAGER and WebGestaltR results to be supported by PubMed literature citations, and we ranked them based on the PubMed score (Table 4; Supplementary Table S4). Each of the six consensus pathways between EnrichR and WebGestaltR also had at least one literature citation support (Supplementary Table S5).

**TABLE 2:** The 33 consensus pathways among PAGER, EnrichR, and WebGestaltR results with PubMed literature support. W vs. P represents the term similarities between WebGestaltR and PAGER results. P vs. E represents the term similarities between PAGER and EnrichR results. W vs. E represents the term similarities between WebGestaltR and EnrichR. k represents the citations of “melanoma” and the keywords from a pathway. OR represents the odds ratio. Score represents the PubMed score . **PMID** represents one PubMed ID example from each entry. BEERE validation represents the semantic relationships retrieved. 1 stands for Yes, and 0 stands for No. All these abbreviations are applied to Table 3 and Table 4.

Term	W vs. P (%)	P vs. E (%)	W vs. E (%)	Keywords	k	OR	Score	PMID	BEERE validation
Photodynamic therapy-induced ap-1 survival signaling.	100	100	100	Photodynamic therapy	1,076	1.150	1.20E+01	31378787	1
mir-509-3p alteration of yap1/ecm axis	100	100	100	mir-509-3p	3	2.376	1.94E+00	33968718	1
Transcriptional misregulation in cancer	100	100	100	Transcriptional misregulation in cancer	10	1.261	1.27E+00	32079144	1
Photodynamic therapy-induced nf-kb survival signaling	100	100	100	Photodynamic, nf-kb	2	1.261	7.40E-01	16524427	1
Apoptosis-related network due to altered notch3 in ovarian cancer	100	100	100	Notch3 ovarian cancer	2	1.154	6.49E-01	28165469	1
Senescence and autophagy in cancer	100	100	100	Senescence and autophagy in cancer	35	0.722	1.97E-02	12789281	1
Focal adhesion: pi3k-akt-mtor-signaling pathway	96	96	100	pi3k-akt-mtor-signaling pathway	36	0.699	1.04E-02	31370278	0
Cytokine-cytokine receptor interaction	100	100	100	Cytokine-cytokine receptor	14	0.442	1.72E-04	34824546	1
il-18 signaling pathway	100	100	100	il-18 pathway	25	0.482	1.54E-05	31731729	1
Mirna targets in ecm and membrane receptors	100	100	100	mirna membrane receptors	2	0.104	1.22E-07	34680340	0
c-type lectin receptor signaling pathway	100	100	100	c-type lectin receptor signaling pathway	28	0.360	4.03E-11	29497419	1
Nod-like receptor signaling pathway	100	100	100	Nod-like receptor signaling pathway	50	0.394	5.13E-15	34747716	0
il-17 signaling pathway	100	100	100	il-17 pathway	20	0.215	2.80E-20	30079767	1
Protein digestion and absorption	100	100	100	Protein digestion and absorption	5	0.062	4.10E-29	30900145	0
Assembly of collagen fibrils and other multimeric structures	100	100	100	Collagen assembly	13	0.126	2.30E-29	29216889	1
Bladder cancer	100	100	100	Bladder cancer	1815	0.708	6.34E-53	35059301	1
Class a/1 (rhodopsin-like receptors)	100	100	100	Adenosine a1 receptor	10	0.056	8.79E-63	8463264	1
Legionellosis	100	100	100	Legionellosis	1	0.006	7.10E-77	17870669	0
Prostaglandin synthesis and regulation	100	100	100	Prostaglandin synthesis and regulation	75	0.162	5.42E-110	3149408	1
Response to elevated platelet cytosolic ca2+	100	100	100	Platelet, calcium	44	0.105	1.95E-120	32562975	1
Hepatitis c and hepatocellular carcinoma	100	100	100	Hepatitis c and hepatocellular carcinoma	20	0.054	4.73E-127	31538700	0
Interleukin-6 family signaling	100	100	100	il-6 signaling pathway	170	0.237	2.42E-131	22713796	1
tnf signaling pathway	100	100	100	tnf signaling pathway	246	0.260	1.65E-159	30591049	1
Inflammatory response pathway	100	100	100	Inflammatory response pathway	123	0.173	2.30E-161	32517213	1
Amoebiasis	100	100	100	Amoebiasis	5	0.012	2.14E-177	31173190	0
Pertussis	100	100	100	Pertussis	83	0.083	1.46E-303	23737697	1
Cytokines and inflammatory response	100	100	100	Cytokines, inflammatory response	559	0.212	0.00E+00	31176707	0
Lung fibrosis	100	100	100	Lung fibrosis	118	0.057	0.00E+00	31249780	1
Malaria	100	100	100	Malaria	137	0.044	0.00E+00	14657217	1
Micrornas in cancer	100	100	100	Micrornas	1,211	0.342	0.00E+00	28118616	1
Rheumatoid arthritis	100	100	100	Rheumatoid arthritis	357	0.074	0.00E+00	27307502	0
*salmonella* infection	100	100	100	*salmonella*	168	0.057	0.00E+00	11773163	0
Spinal cord injury	100	100	100	Spinal cord injury	82	0.034	0.00E+00	30008656	0

**TABLE 3:** The 23 consensus pathways between PAGER, EnrichR results with PubMed literature support.

Term	P vs. E (%)	Keywords	k	OR	Score	PMID	BEERE validation
axl signaling pathway	86	axl signaling	45	1.533	5.30E+00	31871265	0
g alpha (i) signaling events	97	g protein alpha signaling events	15	0.699	6.33E-02	33588787	1
Vitamin d receptor pathway	100	Vitamin d receptor pathway	23	0.734	5.49E-02	28218743	0
Age-rage signaling pathway in diabetic complications	100	Age-rage signaling pathway, diabetes	1	0.200	7.12E-03	25909054	0
Activation of nlrp3 inflammasome by sars-cov-2	100	Viral protein interaction, cytokine receptor	154	0.580	8.48E-14	26920710	0
Viral protein interaction with cytokine and cytokine receptor	100	nlrp3 inflammasome	14	0.225	6.84E-14	33649199	1
pi3k-akt signaling pathway	100	pi3k-akt signaling pathway	475	0.693	2.33E-17	22453015	0
Jak-stat signaling pathway	100	Jak-stat signaling pathway	103	0.478	1.39E-17	32194688	0
Kaposi sarcoma-associated herpesvirus infection	100	Kaposi sarcoma-associated herpesvirus infection	12	0.085	2.15E-45	16443048	0
Proteoglycans in cancer	100	Proteoglycans	766	0.554	8.83E-72	31140988	0
Hematopoietic cell lineage	100	Hematopoietic cell lineage	63	0.130	3.82E-128	26391013	0
Adipogenesis	100	Adipogenesis	25	0.060	1.48E-139	27216185	0
nf-kappa b signaling pathway	100	nf-kappa b signaling	432	0.341	1.46E-158	22433222	1
Glucocorticoid receptor pathway	100	Glucocorticoid receptor	131	0.168	1.91E-179	31911848	1
Gastrin signaling pathway	100	Gastrin	23	0.034	3.80E-246	1,6242076	1
Allograft rejection	100	Allograft rejection	76	0.081	2.85E-288	26951628	0
Human papillomavirus infection	100	Papillomavirus	405	0.237	6.17E-308	10767787	1
Selenium micronutrient network	100	Selenium	129	0.112	2.82e-318	23470450	1
Nanomaterial-induced inflammasome activation	100	Nanotechnology	563	0.160	0.00E+00	28303522	0
Covid-19 adverse outcome pathway	100	Covid-19	289	0.043	0.00E+00	32734626	0
Pathogenic *Escherichia coli* infection	100	*Escherichia coli*	431	0.033	0.00E+00	34912719	0
Lipid and atherosclerosis	100	Lipid, atherosclerosis	21	0.012	0.00E+00	29903879	1
Human cytomegalovirus infection	100	Cytomegalovirus	195	0.125	0.00E+00	15922119	1

**TABLE 4:** The 5 consensus pathways between PAGER, WebGestaltR results with PubMed literature support.

Term	W vs. P (%)	Keywords	k	OR	Score	PMID	BEERE validation
Binding and uptake of ligands by scavenger receptors	100	Ligands, scavenger receptors	12	0.398	6.77E-05	31244937	0
Interleukin-4 and interleukin-13 signaling	100	Interleukin-4, interleukin-13	9	0.114	5.68E-24	23972995	1
Collagen chain trimerization	100	Collagen chain	153	0.257	4.14E-102	21853302	0
Interleukin-10 signaling	100	Interleukin-10	349	0.332	1.05E-136	7852279	1
Post-translational protein phosphorylation	100	Protein phosphorylation	2,850	0.301	0	17973544	0

**FIGURE 4:**
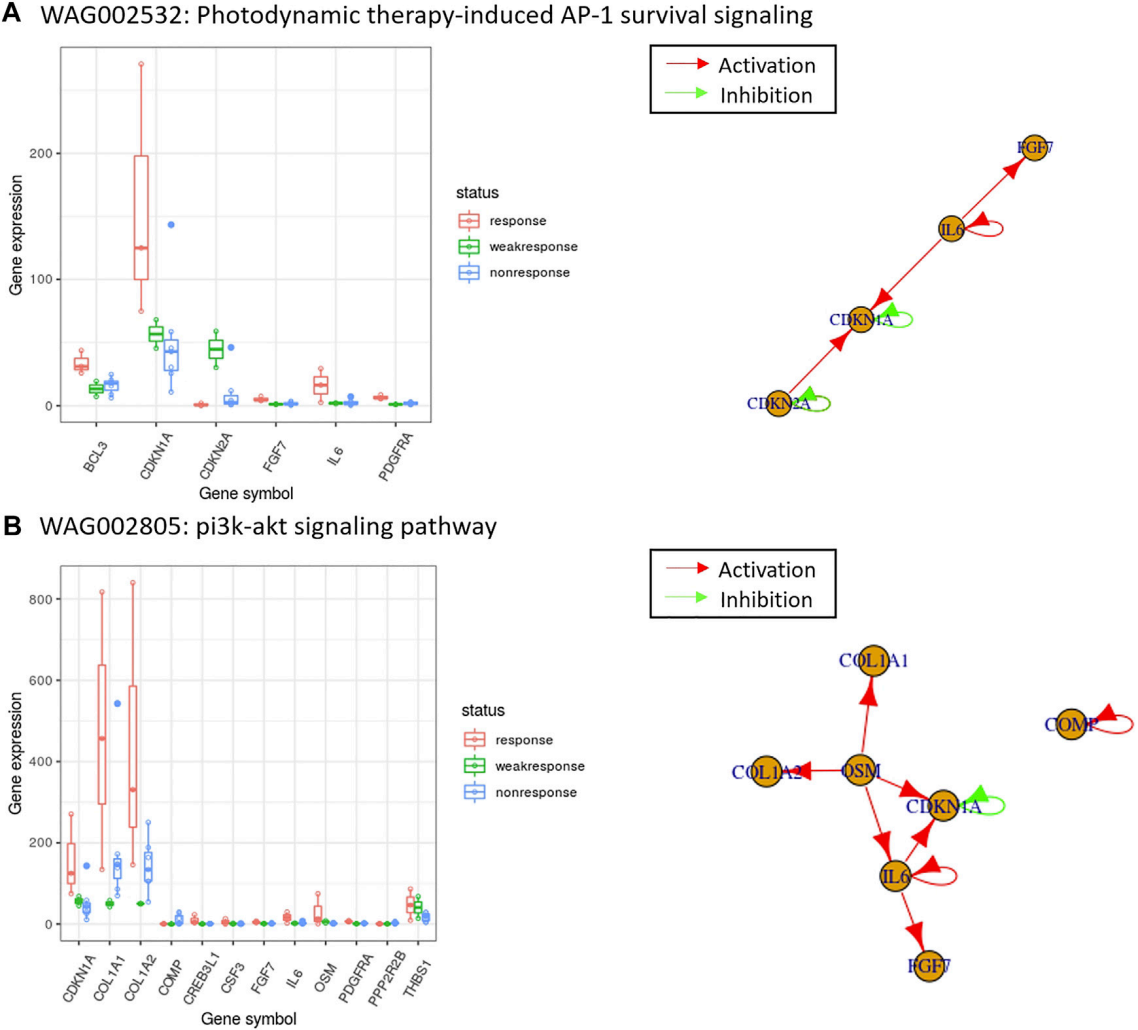
The top-ranked enriched pathways using the PubMed score and the expression of those overlapped genes with gene regulatory networks for melanoma drug resistant-sensitive patients. In the box plots, the *x*-axis are the overlapped genes between differentially expressed gene candidates and pathway gene members, and the *y*-axis are the gene expression values. In the gene regulatory networks, a red arrow indicates the direction of activation, and a green arrow indicates the direction of inhibition. WAG002532 and WAG002805 are the PAG IDs of the enriched pathways shown in **(A)** The pathway with the highest PubMed score in Table 2, and **(B)** one of the PubMed literature validated pathways in Table 3. The details of pathways shown can be retrieved online from: http://discovery.informatics.uab.edu/PAGER/index.php/geneset/view/[PAG ID].

We ranked the pathways based on the PubMed score (Yue et al., 2019a). As reported the highest PubMed score in Table 2, the photodynamic therapy has been frequently reported for melanoma treatment in recent years (Shivashankarappa and Sanjay, 2019; Turkoglu et al., 2019; Abramova et al., 2021; Yordi et al., 2021). We observed that in the overlapped genes between differentially expressed gene candidates and the photodynamic therapy-induced ap-1 survival signaling’s gene members, the five genes, IL6 (Interleukin 6), CDKN1A (Cyclin Dependent Kinase Inhibitor 1A), FGF7 (Fibroblast Growth Factor 7), BCL3 (B-Cell Lymphoma 3-Encoded Protein) and PDGFRA (Platelet Derived Growth Factor Receptor Alpha) were under-expressed in the drug-resistant patients, and the three genes, IL6, CDKN1A and FGF7 were connected in the gene regulatory network from PAGER (de Waal Malefyt et al., 1991) (Figure 4). The pathway, “pi3k-akt signaling pathway” in Table 3, contained twelve overlapped genes. Similarly, among the under-expressed genes, the six genes, IL6 (Interleukin 6), CDKN1A (Cyclin Dependent Kinase Inhibitor 1A), FGF7 (Fibroblast Growth Factor 7), OSM (Oncostatin M), COL1A1 (Collagen Type I Alpha 1 Chain) and COL1A2 (Collagen Type I Alpha 2 Chain) were connected in the gene regulatory network (Figure 4). OSM gene is upstream and stimulates the other five genes. Since OSM is an interleukin-6 (IL-6) type cytokine to inhibit melanoma proliferation, the loss of OSM gene expression in drug-resistant patients may inhibit the activity of collagen biosynthesis and interleukin-6 family signaling. Lacreusette A et al. (Lacreusette et al., 2007) reported that the histone deacetylase inhibitor (HDACi) Trichostatin A (TSA), increased OSM protein activity and histone acetylation of the OSM receptor-beta (OSMRbeta) promoter as well as expression of OSMRbeta mRNA and protein. Therefore, Trichostatin A (TSA) allows the OSM protein to activate the signal transducer and activator of transcription 3 (STAT3) and inhibit proliferation. Thus, OSM/IL-6 resistance of melanoma cells in the late-stage patients may benefit from histone deacetylase inhibitor Trichostatin A.

Another intriguing pathway, the interleukin 10 (IL-10) signaling pathway, reported in PAGER also shows how literature supports its involvement in melanoma. IL-10’s role in immune system biology is that it acts as an immunomodulator, which means that it regulates how the immune system behaves (Terai et al., 2012). Terai et al. found that metastatic melanoma cells can produce IL-10 and that this product can prevent the immune cells from attacking it (Terai et al., 2012). The group also found that IL-6 may play a role in the stimulation of IL-10 production in melanoma cells (Terai et al., 2012). Thus, the PAGER analysis can help give hints to researchers as far as finding potential disease mechanisms is concerned.

We applied precision to measure the performance among the three tools using different cutoffs (Table 5). To evaluate the co-citation coverage in the literature, we tested the result’s precision using different cutoffs. When we set the co-citation ( k ) cutoff to be 1, PAGER’s precision is 0.95 as a little lower than WebGestalt’s precision is 0.99. When the odds ratio cutoff is set to be 0.1, PAGER has the best precision, which is 0.75, and when the PubMed score cutoff is set to be 10e-5, PAGER still leads, giving precision to be 0.30.

**TABLE 5:** The performance of the three tools. k represents the citations of “melanoma” and the keywords from a pathway. OR represents the odds ratio. Score represents the PubMed score .

Tool	Precision		
	*k* > 0	OR > 0.1	score > 10e-5
PAGER	0.95	0.75	0.30
EnrichR	0.89	0.65	0.29
WebGestalt	0.99	0.70	0.24

### Validation of the Enriched Pathways Using the Topology-Based Method and Literature Support in Multiple Sclerosis (MS), Colonic Mucosa in Crohn’s Disease (CD), and Ulcerative Colitis (UC) Studies

**FIGURE 5:**
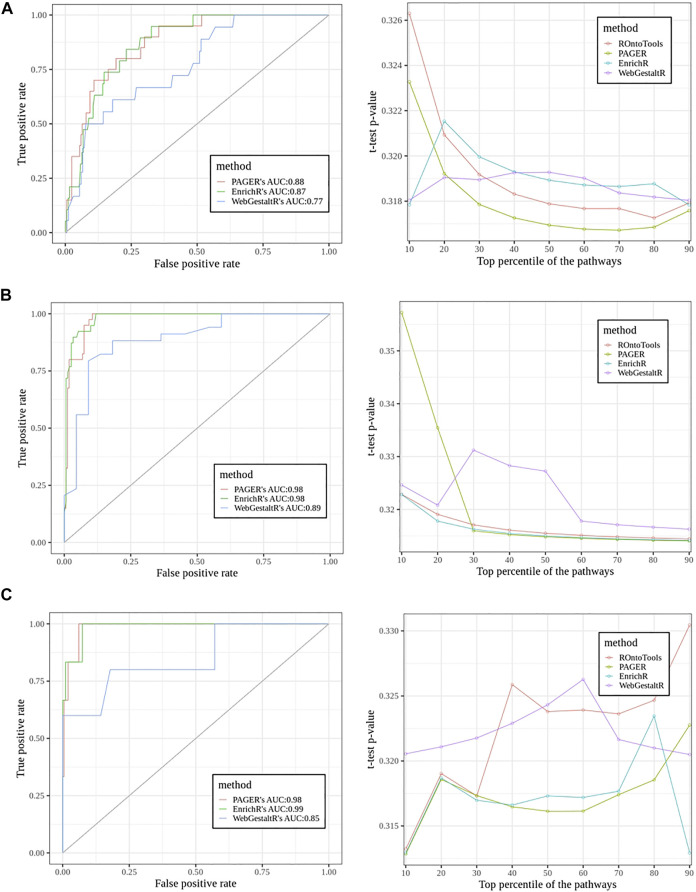
The performance comparisons among PAGER, EnrichrR and WebGestaltR using Receiver Operator Characteristic (ROC) curve and the t-test curve. The pathways’ adjusted *p*-values were applied to generate the ROC curves. The PubMed scores were used for the t-test curve. **(A)** The sclerosis study (E-GEOD-21942). **(B)** The inflamed colonic mucosa vs. non-inflamed colonic mucosa in Crohn’s disease study. **(C)** The inflamed colonic mucosa vs. non-inflamed colonic mucosa in the ulcerative colitis study.

In the sclerosis study, we found 20 pathways in the true set and 203 pathways in the false set using ground truth discovered by the topology-based method, ROntoTools (Figure 5A). The PAGER led by giving the AUC 0.88, EnrichR came the next with AUC to be 0.87, and the WebGestaltR’s AUC was 0.77. In the t-test curve, We found PAGER had the lowest average *p*-value (0.318) compared with ROntoTools (0.319), EnrichR (0.319) and WebgestaltR (0.319). In the inflamed colonic mucosa vs. non-inflamed colonic mucosa in Crohn’s disease study (Figure 5B), we found 40 pathways in the true set and 161 pathways in the false set. Both EnrichR and PAGER had the highest AUC of 0.98, and the WebGestaltR’s AUC was 0.87. We found EnrichR had the lowest average *p*-value (0.316) compared with ROntoTools (0.317), PAGER (0.322) and WebgestaltR (0.322). In the inflamed colonic mucosa vs. non-inflamed colonic mucosa in the ulcerative colitis study (Figure 5C), we found 6 pathways in the true set and 199 pathways in the false set. The EnrichR had the highest AUC of 0.99, PAGER came the next with AUC to be 0.98, and the WebGestaltR’s AUC was 0.85. We found PAGER and EnrichR tied with the lowest average *p*-value (0.317) compared with ROntoTools (0.321) and WebgestaltR (0.322). Overall, PAGER was among the best.

## Discussion and Conclusion

To summarize, we developed an interactive online functional genomics analysis tool, PAGER Web APP. The tool can provide new and significant insights into functional genomics studies and may support precision medicine in delivering the candidate targets. In the melanoma drug-resistant-sensitive case study, we observed that the P-type PAGs (pathways) reported in PAGER lead to insights into molecular mechanisms validated in literature support. PAGER web server supports the feature of r-type PAG-to-PAG network generation.

There are two potential explanations for the differences in the enrichment results among the three tools. First, we noticed that the database versions might vary. As reported in the EnrichR and PAGER Web APP, the KEGG data was processed in 2021, and the WebgestaltR’s KEGG data was processed in 2018. Newer database processing time may suggest more freshly updated content of databases—variability that we couldn’t control in this case study. Second, the enrichment algorithms used in these three tools are different. PAGER adapts hypergeometric test to perform the enrichment analysis and applies adjusted *p*-value using $p_{0}*\;\left(m\;+\;\;n_{p_{i}\leq p_{0}}\;-\;1\right)$ , where $p_{0}$ is the original *p*-value, m is the number of *p*-value’s multiple tests from the PAGs under the constraints of PAG source, overlaps, PAG size, and similarity score, and the $n_{p_{i}\leq p_{0}}$ is the number of *p*-values in the multiple test that has less than or equal to the original *p*-value. EnrichR uses fuzzy enrichment analysis, and applies Benjamini–Hochberg for FDR, according to the documentation online. The WebgestaltR uses hypergeometric test to evaluate the significance of enrichment and uses Bonferroni for *p*-value adjustment. To construct the ground truth in assessing the performance of functional genomics analysis tools, many data-driven approaches can be applied, such as target pathway (Tarca et al., 2012; Tarca et al., 2013) or gene knockout (KO) dataset (Nguyen et al., 2019). In the target pathway approach, the datasets from diseases have a pathway describing the underlying mechanisms, and hence this pathway is implicated in this phenotype. Therefore, a pathway analysis method is assessed based on the ranking and significance of these target pathways. We explored the feasibility of using either pathway ground truth or gene knockout (KO) data sets for our case study, i.e., the study of late-stage drug-resistant melanoma. However, we could not find “target pathway” (Tarca et al., 2012; Tarca et al., 2013) as ground truth or pathways that are not directly related to the dataset to build all the counts in a confusion table. For genes discovered in the enriched pathway, the “pi3k-akt signaling pathway”, we didn’t find any OSM gene-KO Melanoma dataset in the GEO database. We also could not use non-melanoma KO experiments for fear of introducing additional noises. Thus, we used BEERE to extract those semantic relationships that co-mention melanoma and the pathways’ keywords with a statistical evaluation to assess the statistical significance of the PubMed literature reference count above a random model. As for NCBI e-utils literature retrieval, we also applied the PubMed score to evaluate the statistical significance of the literature counts to conquer the literature volume and breadth.

In the future, we expect to implement features to enhance the usage of PAGER Web APP, which can be plugged in geneset, network, and pathway analysis (GNPA) to improve the use. In the current version, we observed that the user interface, especially, in the enriched results, the enriched PAGs’ result is not that interactive enough for users to select a certain number of PAGs or arbitrarily remove some of the records to generate PAG-to-PAG networks. We will implement the interactive panels in the future release. PAGER Web APP calls the PAGER API, which implements an over-representation analysis (ORA) technique by default. In general, advanced functional class scoring (FCS) techniques, e.g., Gene Set Enrichment Analysis (GSEA) (Subramanian et al., 2005), Gene Set Analysis (GSA) (Efron and Tibshirani, 2007) and Pathway Analysis with Down-weighting of Overlapping Genes (PADOG) (Tarca et al., 2012), can better detect the significant effects on pathways led by large changes in individual genes and the weaker coordinated. Other pathway analysis tools may also incorporate network topology information to integrate signaling interactions among genes in a pathway, e.g., Pathway-Express (Khatri et al., 2007), SPIA (Tarca et al., 2009), Pathway-Guide (Advaita Bioinformatics, http://www.advaitabio.com), TopoGSA (Glaab et al., 2010), Bayesian Pathway Analysis (BPA) (Isci et al., 2011), and PathNet (Dutta et al., 2012), etc. We plan to implement additional advanced topology-based pathway GSEA analysis techniques into the PAGER APIs, and adopt comprehensive benchmark data sets (Tarca et al., 2012; Tarca et al., 2013; Nguyen et al., 2019) to guide users in selecting the proper method for the right application scenario in future releases. Thus, PAGER Web APP will offer users more expanded analysis choices than today.

## Data Availability

The original contributions presented in the study are included in the article/Supplementary Material, further inquiries can be directed to the corresponding author.
