# Supplementary material for: PAGER Web APP: An Interactive, Online Gene Set and Network Interpretation Tool for Functional Genomics
Source: Front Genet. 2022 Apr 12;13:820361. doi: 10.3389/fgene.2022.820361 (PMC9039620; doi:10.3389/fgene.2022.820361)
Supplement: Supplementary file 1 [file DataSheet1.docx]

Supplementary Material

# Supplementary Tables

Supplementary Table 1. The enriched P-type PAGs in PAGER. OLAP represents the number of overlapped genes between the PAG members and candidate genes.

| **ID** | MEAN | **NAME** | **SOURCE** | **OLAP** | **SIMILARITY_SCORE** | **P-VALUE** | **FDR** |
| --- | --- | --- | --- | --- | --- | --- | --- |
| WAG002732 | -3.12 | Interleukin-4 and Interleukin-13 signaling | WikiPathway_2021 | 14 | 0.0594 | 1.23E-15 | 2.40E-12 |
| WAG003238 | -2.71 | Network map of SARS-CoV-2 signaling pathway | WikiPathway_2021 | 17 | 0.0451 | 1.23E-14 | 2.23E-11 |
| WAG003031 | -2.96 | IL-18 signaling pathway | WikiPathway_2021 | 18 | 0.0436 | 2.09E-14 | 3.79E-11 |
| WAG003196 | -2.62 | Burn wound healing | WikiPathway_2021 | 13 | 0.0551 | 3.47E-14 | 6.73E-11 |
| WIG001524 | -2.25 | Akt Signaling | Protein Lounge | 20 | 0.0422 | 1.01E-13 | 1.77E-10 |
| WIG001643 | -2.98 | Antioxidant Action of Vitamin-C | Protein Lounge | 19 | 0.0409 | 3.83E-13 | 6.69E-10 |
| WIG001614 | -3.00 | PAK Pathway | Protein Lounge | 16 | 0.0396 | 1.21E-12 | 2.10E-09 |
| WIG001627 | -2.84 | PI3K Signaling | Protein Lounge | 22 | 0.0401 | 1.49E-12 | 2.59E-09 |
| WIG001660 | -2.76 | Rho Family GTPases | Protein Lounge | 18 | 0.0395 | 1.74E-12 | 3.03E-09 |
| WAG002537 | -3.37 | Lung fibrosis | WikiPathway_2021 | 10 | 0.0587 | 2.08E-12 | 4.06E-09 |
| WAG001820 | -4.62 | hsa04657 IL-17 signaling pathway - Homo sapiens (human) | KEGG_2021_HUMAN | 11 | 0.0503 | 5.06E-12 | 9.28E-09 |
| WIG001680 | -3.91 | STAT3 Pathway | Protein Lounge | 14 | 0.0399 | 7.83E-12 | 1.36E-08 |
| WAG003220 | -4.57 | Overview of proinflammatory and profibrotic mediators | WikiPathway_2021 | 12 | 0.0454 | 7.71E-12 | 1.40E-08 |
| WIG001752 | -2.65 | Molecular Mechanisms of Cancer | Protein Lounge | 21 | 0.0383 | 1.08E-11 | 1.86E-08 |
| WAG003276 | -1.88 | Senescence and autophagy in cancer | WikiPathway_2021 | 11 | 0.047 | 1.72E-11 | 3.16E-08 |
| WIG001556 | -4.87 | all-trans-Retinoic Acid Signaling in Brain | Protein Lounge | 11 | 0.0464 | 2.12E-11 | 3.88E-08 |
| WIG001741 | -4.87 | MIF Mediated Glucocorticoid Regulation | Protein Lounge | 11 | 0.0457 | 2.87E-11 | 5.23E-08 |
| WIG001688 | -2.63 | Tec Kinases Signaling | Protein Lounge | 16 | 0.0365 | 3.60E-11 | 6.00E-08 |
| WIG001742 | -4.87 | MIF Regulation of Innate Immune Cells | Protein Lounge | 11 | 0.0452 | 3.50E-11 | 6.35E-08 |
| WAG001827 | -3.72 | hsa04668 TNF signaling pathway - Homo sapiens (human) | KEGG_2021_HUMAN | 11 | 0.0452 | 3.50E-11 | 6.36E-08 |
| WIG001488 | -2.55 | ERK Signaling | Protein Lounge | 17 | 0.0359 | 7.89E-11 | 1.31E-07 |
| WAG001907 | -1.90 | hsa05130 Pathogenic Escherichia coli infection - Homo sapiens (human) | KEGG_2021_HUMAN | 13 | 0.0375 | 8.62E-11 | 1.49E-07 |
| WAG001753 | -3.85 | hsa04060 Cytokine-cytokine receptor interaction - Homo sapiens (human) | KEGG_2021_HUMAN | 15 | 0.0352 | 1.18E-10 | 1.94E-07 |
| WIG001562 | -3.87 | IL-6 Pathway | Protein Lounge | 12 | 0.0387 | 1.51E-10 | 2.61E-07 |
| WIG001622 | -4.03 | PEDF Induced Signaling | Protein Lounge | 11 | 0.0414 | 1.62E-10 | 2.84E-07 |
| WAG002729 | -4.52 | Interleukin-10 signaling | WikiPathway_2021 | 8 | 0.0575 | 1.62E-10 | 3.16E-07 |
| WIG001833 | -2.11 | Hearing and Vision Proteins | Spike | 16 | 0.0345 | 2.98E-10 | 4.88E-07 |
| WIG001440 | -4.83 | Cellular Immune Responses to HBV | Protein Lounge | 10 | 0.0422 | 4.52E-10 | 7.91E-07 |
| WAG003213 | -3.14 | Prostaglandin signaling | WikiPathway_2021 | 7 | 0.0632 | 5.27E-10 | 1.03E-06 |
| WAG002536 | -3.84 | Photodynamic therapy-induced NF-kB survival signaling | WikiPathway_2021 | 7 | 0.0606 | 8.21E-10 | 1.61E-06 |
| WIG001529 | -4.07 | Granulocyte Adhesion and Diapedesis | Protein Lounge | 12 | 0.0342 | 1.36E-09 | 2.22E-06 |
| WAG001974 | -2.97 | hsa05417 Lipid and atherosclerosis - Homo sapiens (human) | KEGG_2021_HUMAN | 12 | 0.0326 | 3.06E-09 | 4.96E-06 |
| WAG003063 | -0.49 | AXL signaling pathway | WikiPathway_2021 | 9 | 0.0408 | 3.01E-09 | 5.25E-06 |
| WIG001475 | -4.66 | Endothelin-1 Signaling Pathway | Protein Lounge | 12 | 0.0324 | 3.40E-09 | 5.50E-06 |
| WAG002892 | -4.09 | Class A/1 (rhodopsin-like receptors) | WikiPathway_2021 | 14 | 0.0317 | 3.78E-09 | 5.93E-06 |
| WAG007272 | -2.15 | Gelatin degradation by MMP1, 2, 3, 7, 8, 9, 12, 13 | Reactome_2021 | 6 | 0.0683 | 3.22E-09 | 6.39E-06 |
| WIG001648 | -4.83 | RAR-Gamma/RXR-Alpha Degradation | Protein Lounge | 10 | 0.0367 | 4.06E-09 | 6.97E-06 |
| WAG007806 | -4.94 | IL10 negatively regulates extracellular inflammatory mediators | Reactome_2021 | 6 | 0.0663 | 4.22E-09 | 8.36E-06 |
| WIG001843 | -2.88 | NFkB Signaling Network | Spike | 10 | 0.036 | 5.39E-09 | 8.95E-06 |
| WAG002339 | -2.14 | Assembly of collagen fibrils and other multimeric structures | WikiPathway_2021 | 7 | 0.051 | 5.25E-09 | 9.66E-06 |
| WAG001918 | -3.48 | hsa05146 Amoebiasis - Homo sapiens (human) | KEGG_2021_HUMAN | 9 | 0.039 | 5.65E-09 | 9.79E-06 |
| WIG001779 | -4.52 | Bacterial Meningitis | Protein Lounge | 9 | 0.039 | 5.65E-09 | 9.79E-06 |
| WAG003066 | -3.17 | Endothelin pathway | WikiPathway_2021 | 11 | 0.0319 | 1.19E-08 | 1.92E-05 |
| WAG001916 | -2.58 | hsa05144 Malaria - Homo sapiens (human) | KEGG_2021_HUMAN | 7 | 0.0475 | 1.12E-08 | 2.06E-05 |
| WAG002659 | -1.33 | Focal adhesion: PI3K-Akt-mTOR-signaling pathway | WikiPathway_2021 | 13 | 0.0301 | 1.54E-08 | 2.40E-05 |
| WAG002896 | -3.55 | G alpha (i) signaling events | WikiPathway_2021 | 12 | 0.0296 | 1.85E-08 | 2.87E-05 |
| WAG007292 | -1.99 | FN1 binds Collagen types I-V, VII | Reactome_2021 | 5 | 0.0824 | 1.39E-08 | 2.87E-05 |
| WIG001480 | -4.04 | Agranulocyte Adhesion and Diapedesis | Protein Lounge | 11 | 0.0309 | 1.99E-08 | 3.11E-05 |
| WAG002190 | -2.42 | Spinal cord injury | WikiPathway_2021 | 9 | 0.0351 | 2.52E-08 | 4.15E-05 |
| WAG003512 | -3.75 | The Ligand:GPCR:Gi complex dissociates | Reactome_2021 | 11 | 0.0302 | 2.80E-08 | 4.37E-05 |
| WAG003513 | -3.75 | Liganded Gi-activating GPCRs bind inactive heterotrimeric G-protein Gi | Reactome_2021 | 11 | 0.0302 | 2.80E-08 | 4.37E-05 |
| WAG001911 | -4.21 | hsa05134 Legionellosis - Homo sapiens (human) | KEGG_2021_HUMAN | 7 | 0.0436 | 2.87E-08 | 5.19E-05 |
| WAG003510 | -3.75 | Liganded Gi-activating GPCR acts as a GEF for Gi | Reactome_2021 | 11 | 0.0299 | 3.39E-08 | 5.27E-05 |
| WAG007310 | -1.52 | Syndecan-1 binds collagen types I, III, V | Reactome_2021 | 4 | 0.1272 | 3.10E-08 | 6.75E-05 |
| WAG001965 | -5.27 | hsa05323 Rheumatoid arthritis - Homo sapiens (human) | KEGG_2021_HUMAN | 8 | 0.0367 | 4.91E-08 | 8.42E-05 |
| WAG001813 | -3.76 | hsa04621 NOD-like receptor signaling pathway - Homo sapiens (human) | KEGG_2021_HUMAN | 10 | 0.03 | 8.16E-08 | 0.000127 |
| WIG001691 | -3.20 | TGF-Beta Pathway | Protein Lounge | 10 | 0.0294 | 1.10E-07 | 0.00017 |
| WAG001886 | -3.01 | hsa04974 Protein digestion and absorption - Homo sapiens (human) | KEGG_2021_HUMAN | 8 | 0.0344 | 1.09E-07 | 0.000178 |
| WAG006760 | -3.20 | Expression of STAT3-upregulated extracellular proteins | Reactome_2021 | 5 | 0.063 | 9.11E-08 | 0.000179 |
| WAG001756 | -4.31 | hsa04064 NF-kappa B signaling pathway - Homo sapiens (human) | KEGG_2021_HUMAN | 8 | 0.0342 | 1.17E-07 | 0.000191 |
| WAG003212 | -2.44 | Malignant pleural mesothelioma | WikiPathway_2021 | 14 | 0.0284 | 1.27E-07 | 0.000196 |
| WAG007306 | -1.52 | DDR2 binds collagen type I, II, III, V, X fibrils | Reactome_2021 | 4 | 0.1017 | 1.10E-07 | 0.00023 |
| WIG001523 | -4.11 | Airway Pathology in COPD | Protein Lounge | 7 | 0.0369 | 1.78E-07 | 0.000306 |
| WAG003219 | -2.84 | Orexin receptor pathway | WikiPathway_2021 | 9 | 0.03 | 2.20E-07 | 0.000343 |
| WAG002093 | -0.55 | Response to elevated platelet cytosolic Ca2+ | WikiPathway_2021 | 8 | 0.0323 | 2.37E-07 | 0.000383 |
| WAG001926 | -1.33 | hsa05165 Human papillomavirus infection - Homo sapiens (human) | KEGG_2021_HUMAN | 12 | 0.0269 | 3.28E-07 | 0.000483 |
| WAG003204 | -0.39 | PPAR Beta/Delta pathway | WikiPathway_2021 | 5 | 0.0536 | 2.87E-07 | 0.000555 |
| WAG002532 | -0.91 | Photodynamic therapy-induced AP-1 survival signaling. | WikiPathway_2021 | 6 | 0.0407 | 3.31E-07 | 0.000577 |
| WAG007275 | -1.52 | Formation of collagen fibrils | Reactome_2021 | 4 | 0.0855 | 2.86E-07 | 0.000592 |
| WAG007287 | -1.52 | Formation of collagen fibres | Reactome_2021 | 4 | 0.0855 | 2.86E-07 | 0.000593 |
| WAG002805 | -1.42 | PI3K-Akt signaling pathway | WikiPathway_2021 | 12 | 0.0266 | 4.48E-07 | 0.000657 |
| WAG001924 | -2.67 | hsa05163 Human cytomegalovirus infection - Homo sapiens (human) | KEGG_2021_HUMAN | 10 | 0.0263 | 5.13E-07 | 0.00075 |
| WAG002366 | -1.25 | Apoptosis-related network due to altered Notch3 in ovarian cancer | WikiPathway_2021 | 6 | 0.0391 | 4.70E-07 | 0.000815 |
| WAG003262 | -4.58 | Cytokines and inflammatory response | WikiPathway_2021 | 5 | 0.0494 | 5.09E-07 | 0.000933 |
| WAG001778 | -1.42 | hsa04151 PI3K-Akt signaling pathway - Homo sapiens (human) | KEGG_2021_HUMAN | 12 | 0.0262 | 6.60E-07 | 0.000962 |
| WAG007322 | -1.50 | LAIR2 binds collagen | Reactome_2021 | 3 | 0.1598 | 6.58E-07 | 0.00144 |
| WAG002374 | -1.27 | Vitamin D receptor pathway | WikiPathway_2021 | 9 | 0.0265 | 1.08E-06 | 0.00159 |
| WAG001909 | -2.25 | hsa05132 Salmonella infection - Homo sapiens (human) | KEGG_2021_HUMAN | 10 | 0.0248 | 1.27E-06 | 0.00179 |
| WAG001934 | -1.67 | hsa05202 Transcriptional misregulation in cancer - Homo sapiens (human) | KEGG_2021_HUMAN | 9 | 0.0262 | 1.23E-06 | 0.0018 |
| WIG001806 | -3.90 | C. pneumoniae Infection in Atherosclerosis | Protein Lounge | 5 | 0.045 | 9.96E-07 | 0.00181 |
| WAG001928 | -3.51 | hsa05167 Kaposi sarcoma-associated herpesvirus infection - Homo sapiens (human) | KEGG_2021_HUMAN | 9 | 0.0261 | 1.34E-06 | 0.00195 |
| WIG001678 | -4.37 | SOCS Pathway | Protein Lounge | 8 | 0.028 | 1.38E-06 | 0.00204 |
| WAG001754 | -5.10 | hsa04061 Viral protein interaction with cytokine and cytokine receptor - Homo sapiens (human) | KEGG_2021_HUMAN | 7 | 0.0306 | 1.38E-06 | 0.00215 |
| WAG003083 | -4.85 | COVID-19 adverse outcome pathway | WikiPathway_2021 | 4 | 0.0659 | 1.16E-06 | 0.00229 |
| WAG007281 | -1.52 | Removal of fibrillar collagen N-propeptides | Reactome_2021 | 4 | 0.0659 | 1.16E-06 | 0.00229 |
| WAG001816 | -2.92 | hsa04625 C-type lectin receptor signaling pathway - Homo sapiens (human) | KEGG_2021_HUMAN | 7 | 0.0299 | 1.79E-06 | 0.00278 |
| WAG007282 | -1.52 | Removal of fibrillar collagen C-propeptides | Reactome_2021 | 4 | 0.0625 | 1.54E-06 | 0.00301 |
| WAG007293 | -1.52 | DDR1 binds collagen type I, II, III, IV, V, XI fibrils | Reactome_2021 | 4 | 0.0625 | 1.54E-06 | 0.00301 |
| WAG003325 | 0.05 | FAM20C phosphorylates FAM20C substrates | Reactome_2021 | 7 | 0.0292 | 2.30E-06 | 0.00356 |
| WAG010361 | -1.97 | FPR2 binds FPR2 ligands | Reactome_2021 | 3 | 0.1298 | 1.64E-06 | 0.00356 |
| WAG002771 | 0.05 | Post-translational protein phosphorylation | WikiPathway_2021 | 7 | 0.029 | 2.44E-06 | 0.00378 |
| WAG007309 | -2.09 | Gelatin degradation by MMP19 | Reactome_2021 | 4 | 0.0595 | 2.00E-06 | 0.00391 |
| WIG001651 | -3.67 | Regulation of eIF4 and p70S6K | Protein Lounge | 8 | 0.0264 | 2.74E-06 | 0.004 |
| WAG002378 | -4.38 | Glucocorticoid receptor pathway | WikiPathway_2021 | 6 | 0.0324 | 2.66E-06 | 0.00431 |
| WAG002354 | -0.59 | Bladder cancer | WikiPathway_2021 | 5 | 0.0393 | 2.66E-06 | 0.00461 |
| WAG001817 | -3.39 | hsa04630 JAK-STAT signaling pathway - Homo sapiens (human) | KEGG_2021_HUMAN | 8 | 0.026 | 3.29E-06 | 0.00477 |
| WAG002680 | -0.68 | miR-509-3p alteration of YAP1/ECM axis | WikiPathway_2021 | 4 | 0.0568 | 2.55E-06 | 0.00499 |
| WAG003002 | -3.13 | Gastrin signaling pathway | WikiPathway_2021 | 7 | 0.0282 | 3.28E-06 | 0.00505 |
| WAG002329 | -2.33 | Binding and uptake of ligands by scavenger receptors | WikiPathway_2021 | 5 | 0.0387 | 3.01E-06 | 0.0052 |
| WAG001950 | -0.59 | hsa05219 Bladder cancer - Homo sapiens (human) | KEGG_2021_HUMAN | 5 | 0.0387 | 3.01E-06 | 0.00521 |
| WAG002753 | -1.99 | Collagen chain trimerization | WikiPathway_2021 | 5 | 0.0387 | 3.01E-06 | 0.00521 |
| WIG001608 | -3.92 | p38 Signaling | Protein Lounge | 10 | 0.0237 | 3.78E-06 | 0.00526 |
| WIG001623 | -4.59 | PGC1Alpha Pathway | Protein Lounge | 7 | 0.0279 | 3.68E-06 | 0.00545 |
| WIG001579 | -3.86 | JAK/STAT Pathway | Protein Lounge | 10 | 0.0236 | 4.14E-06 | 0.00576 |
| WAG007285 | -1.99 | Secretion of collagens | Reactome_2021 | 5 | 0.038 | 3.40E-06 | 0.00587 |
| WIG001442 | -1.03 | Actin-Based Motility by Rho Family GTPases | Protein Lounge | 8 | 0.0255 | 4.10E-06 | 0.00594 |
| WAG001910 | -3.45 | hsa05133 Pertussis - Homo sapiens (human) | KEGG_2021_HUMAN | 6 | 0.0311 | 3.95E-06 | 0.00619 |
| WAG006305 | -0.82 | PYCARD recruits procaspase-1 via CARD | Reactome_2021 | 3 | 0.1097 | 3.26E-06 | 0.00708 |
| WAG007204 | -1.50 | OSCAR binds collagen and SP-D | Reactome_2021 | 3 | 0.1097 | 3.26E-06 | 0.00708 |
| WAG007311 | -1.50 | MSR1 (SCARA1) binds collagen | Reactome_2021 | 3 | 0.1097 | 3.26E-06 | 0.00708 |
| WAG001932 | -3.95 | hsa05171 Coronavirus disease - COVID-19 - Homo sapiens (human) | KEGG_2021_HUMAN | 9 | 0.0232 | 5.65E-06 | 0.00783 |
| WAG007330 | -1.99 | P4HB:4-Hyp collagen propeptides dissociates | Reactome_2021 | 5 | 0.0363 | 4.81E-06 | 0.00798 |
| WAG007280 | -1.99 | Glucosylation of collagen propeptide hydroxylysines | Reactome_2021 | 5 | 0.0363 | 4.81E-06 | 0.00799 |
| WAG007326 | -1.99 | PLOD3:Fe2+ dimer:Glucosyl-galactosyl-hydroxylysyl collagen propeptides dissociates | Reactome_2021 | 5 | 0.0363 | 4.81E-06 | 0.00799 |
| WAG007283 | -1.99 | P4HB binds Collagen chains | Reactome_2021 | 5 | 0.0363 | 4.81E-06 | 0.008 |
| WAG007324 | -1.99 | PLOD3 binds Lysyl hydroxylated collagen propeptides | Reactome_2021 | 5 | 0.0363 | 4.81E-06 | 0.008 |
| WAG007279 | -1.99 | Galactosylation of collagen propeptide hydroxylysines by PLOD3 | Reactome_2021 | 5 | 0.0363 | 4.81E-06 | 0.00801 |
| WAG007325 | -1.99 | PLOD3:Fe2+ dimer:Galactosyl-hydroxylysyl collagen propeptides dissociates | Reactome_2021 | 5 | 0.0363 | 4.81E-06 | 0.00801 |
| WAG003308 | -2.18 | Prostaglandin synthesis and regulation | WikiPathway_2021 | 5 | 0.0363 | 4.81E-06 | 0.00802 |
| WAG002386 | -1.74 | miRNA targets in ECM and membrane receptors | WikiPathway_2021 | 5 | 0.0358 | 5.36E-06 | 0.00887 |
| WAG007278 | -1.99 | Galactosylation of collagen propeptide hydroxylysines by procollagen galactosyltransferases 1, 2. | Reactome_2021 | 5 | 0.0358 | 5.36E-06 | 0.00887 |
| WAG007327 | -1.99 | COLGALT1,COLGALT2 bind Lysyl hydroxylated collagen propeptides | Reactome_2021 | 5 | 0.0358 | 5.36E-06 | 0.00888 |
| WAG007328 | -1.99 | COLGALT1,COLGALT2:Galactosyl-hydroxylysyl collagen propeptides dissociates | Reactome_2021 | 5 | 0.0358 | 5.36E-06 | 0.00889 |
| WAG002340 | 0.05 | Regulation of IGF transport and uptake by IGF binding proteins | WikiPathway_2021 | 7 | 0.0265 | 6.32E-06 | 0.00926 |
| WAG007277 | -1.99 | Procollagen lysyl hydroxylases convert collagen lysines to 5-hydroxylysines | Reactome_2021 | 5 | 0.0353 | 5.97E-06 | 0.00986 |
| WAG007329 | -1.99 | Lysyl hydroxylated collagen propeptides dissociate from Lysyl hydroxylases | Reactome_2021 | 5 | 0.0353 | 5.97E-06 | 0.00987 |
| WAG002358 | -1.33 | Pluripotent stem cell differentiation pathway | WikiPathway_2021 | 5 | 0.0348 | 6.63E-06 | 0.0109 |
| WAG005407 | -1.99 | Procollagen triple helix formation | Reactome_2021 | 5 | 0.0348 | 6.63E-06 | 0.0109 |
| WAG007276 | -1.99 | Prolyl 4-hydroxylase converts collagen prolines to 4-hydroxyprolines | Reactome_2021 | 5 | 0.0348 | 6.63E-06 | 0.0109 |
| WIG001640 | -0.52 | PTEN Pathway | Protein Lounge | 8 | 0.024 | 7.95E-06 | 0.0111 |
| WAG001938 | -0.89 | hsa05206 MicroRNAs in cancer - Homo sapiens (human) | KEGG_2021_HUMAN | 10 | 0.0229 | 8.50E-06 | 0.0116 |
| WAG002179 | -0.76 | Adipogenesis | WikiPathway_2021 | 7 | 0.0259 | 8.13E-06 | 0.0118 |
| WAG002629 | -3.11 | Nanomaterial-induced inflammasome activation | WikiPathway_2021 | 3 | 0.0954 | 5.67E-06 | 0.0118 |
| WAG003077 | -3.11 | Activation of NLRP3 inflammasome by SARS-CoV-2 | WikiPathway_2021 | 3 | 0.0954 | 5.67E-06 | 0.0118 |
| WAG007284 | -1.68 | Formation of allysine by LOX | Reactome_2021 | 3 | 0.0954 | 5.67E-06 | 0.0118 |
| WAG007301 | -1.68 | Formation of hydroxyallysine by LOX | Reactome_2021 | 3 | 0.0954 | 5.67E-06 | 0.0118 |
| WAG005406 | -1.99 | Collagen prolyl 3-hydroxylase converts 4-Hyp collagen to 3,4-Hyp collagen | Reactome_2021 | 5 | 0.0343 | 7.34E-06 | 0.012 |
| WAG005408 | -1.99 | Prolyl 3-hydroxylases:Fe2+:3,4-Hyp collagen propeptides dissociates | Reactome_2021 | 5 | 0.0343 | 7.34E-06 | 0.012 |
| WAG005409 | -1.99 | P3HB binds 4-Hyp-collagen propeptides | Reactome_2021 | 5 | 0.0343 | 7.34E-06 | 0.012 |
| WIG001522 | -4.22 | Glucocorticoid Receptor Signaling | Protein Lounge | 8 | 0.0239 | 8.59E-06 | 0.012 |
| WAG003150 | -2.70 | Cell recruitment (pro-inflammatory response) | WikiPathway_2021 | 4 | 0.0469 | 7.19E-06 | 0.0132 |
| WIG001590 | -5.14 | NFAT Signaling and Lymphocyte Interactions | Protein Lounge | 6 | 0.0282 | 9.79E-06 | 0.0145 |
| WIG001712 | -3.80 | Transendothelial Migration of Leukocytes | Protein Lounge | 9 | 0.0222 | 1.08E-05 | 0.0146 |
| WAG002000 | -3.94 | Selenium micronutrient network | WikiPathway_2021 | 6 | 0.028 | 1.04E-05 | 0.0155 |
| WAG002782 | -3.52 | Interleukin-6 family signaling | WikiPathway_2021 | 4 | 0.0455 | 8.59E-06 | 0.0156 |
| WAG002718 | -2.23 | Neutrophil degranulation | WikiPathway_2021 | 12 | 0.0234 | 1.22E-05 | 0.0169 |
| WAG003202 | -1.01 | PPAR-gamma pathway | WikiPathway_2021 | 4 | 0.0441 | 1.02E-05 | 0.0184 |
| WAG012362 | -4.98 | Expression of IL4,IL13-downregulated extracellular genes | Reactome_2021 | 3 | 0.0846 | 9.02E-06 | 0.0186 |
| WAG007304 | 0.94 | COMP binds collagen, fibronectin, aggrecan and matrilins | Reactome_2021 | 3 | 0.0846 | 9.02E-06 | 0.0187 |
| WAG002173 | -2.30 | Allograft Rejection | WikiPathway_2021 | 6 | 0.0272 | 1.34E-05 | 0.0197 |
| WAG003247 | -1.47 | Alzheimer's disease | WikiPathway_2021 | 9 | 0.0218 | 1.51E-05 | 0.0202 |
| WIG001689 | 0.51 | Telomerase Components in Cell Signaling | Protein Lounge | 7 | 0.0244 | 1.49E-05 | 0.021 |
| WAG002548 | -2.73 | Hepatitis C and hepatocellular carcinoma | WikiPathway_2021 | 5 | 0.0314 | 1.42E-05 | 0.0222 |
| WAG001937 | -1.49 | hsa05205 Proteoglycans in cancer - Homo sapiens (human) | KEGG_2021_HUMAN | 8 | 0.0223 | 1.78E-05 | 0.024 |
| WIG001635 | 0.41 | PPAR Pathway | Protein Lounge | 7 | 0.0239 | 1.85E-05 | 0.0259 |
| WAG001818 | -3.33 | hsa04640 Hematopoietic cell lineage - Homo sapiens (human) | KEGG_2021_HUMAN | 6 | 0.0264 | 1.79E-05 | 0.0262 |
| WIG001733 | -1.26 | MAPK Signaling | Protein Lounge | 10 | 0.0221 | 1.94E-05 | 0.0262 |
| WAG001871 | -3.24 | hsa04933 AGE-RAGE signaling pathway in diabetic complications - Homo sapiens (human) | KEGG_2021_HUMAN | 6 | 0.0262 | 1.89E-05 | 0.0276 |
| WAG011578 | -5.74 | Receptor CXCR2 binds ligands CXCL1 to 7 | Reactome_2021 | 3 | 0.0763 | 1.34E-05 | 0.0278 |
| WIG001655 | -0.21 | Renin-Angiotensin Pathway | Protein Lounge | 8 | 0.0219 | 2.18E-05 | 0.0292 |
| WAG004994 | -1.52 | PTK2 binds activated MET | Reactome_2021 | 4 | 0.0395 | 1.87E-05 | 0.0324 |
| WIG001838 | -2.47 | Caspases Cascade | Spike | 6 | 0.0256 | 2.36E-05 | 0.0342 |
| WAG007294 | -1.50 | DCN binds collagen I, II, III, VI fibrils | Reactome_2021 | 3 | 0.0696 | 1.91E-05 | 0.0379 |
| WAG002960 | -1.51 | Inflammatory response pathway | WikiPathway_2021 | 4 | 0.0376 | 2.45E-05 | 0.0422 |
| WAG003236 | -5.41 | Antiviral and anti-inflammatory effects of Nrf2 on SARS-CoV-2 pathway | WikiPathway_2021 | 4 | 0.0368 | 2.78E-05 | 0.0478 |
| WAG001933 | -0.90 | hsa05200 Pathways in cancer - Homo sapiens (human) | KEGG_2021_HUMAN | 12 | 0.0224 | 3.56E-05 | 0.0482 |
| WIG001597 | 0.41 | Nuclear Receptor Activation by Vitamin-A | Protein Lounge | 7 | 0.0223 | 3.66E-05 | 0.0494 |
| WAG002284 | -1.99 | Collagen biosynthesis and modifying enzymes | WikiPathway_2021 | 5 | 0.028 | 3.37E-05 | 0.05 |
| WAG003309 | -1.27 | Exocytosis of platelet alpha granule contents | Reactome_2021 | 5 | 0.028 | 3.37E-05 | 0.05 |

Supplementary Table 2. Validation of consensus pathways among PAGER, EnrichR, and WebGestaltR results using BEERE. Score represented the RDS score in BEERE. S2O_CNT represents the number of literature jointly mention subject to object semantic relationship. All these abbreviations are applied to Supplementary table 3 and Supplementary table 4.

| **Term** | **Keywords** | **Subject** | **Object** | **Score** | **Predicate** | **S2O_CNT** |
| --- | --- | --- | --- | --- | --- | --- |
| apoptosis-related network due to altered notch3 in ovarian cancer | apoptosis | melanoma cell | apoptosis | 929.12 | LOCATION_OF | 580 |
| apoptosis-related network due to altered notch3 in ovarian cancer | apoptosis | apoptosis | melanoma | 730.73 | PROCESS_OF | 191 |
| lung fibrosis | lung | melanoma cell | secondary malignant neoplasm of lung | 449.12 | LOCATION_OF | 97 |
| senescence and autophagy in cancer | autophagy | autophagy | melanoma | 150.64 | PROCESS_OF | 36 |
| apoptosis-related network due to altered notch3 in ovarian cancer | apoptosis | apoptosis | melanoma cell | 73.32 | NEG_AFFECTS | 31 |
| transcriptional misregulation in cancer | transcriptional | transcriptional regulation | melanoma | 52.60 | PROCESS_OF | 13 |
| senescence and autophagy in cancer | autophagy | melanoma cell | autophagy | 47.60 | LOCATION_OF | 30 |
| lung fibrosis | lung | melanoma | secondary malignant neoplasm of lung | 45.22 | CAUSES | 9 |
| apoptosis-related network due to altered notch3 in ovarian cancer | apoptosis | apoptosis | melanoma cell | 42.99 | AFFECTS | 24 |
| apoptosis-related network due to altered notch3 in ovarian cancer | apoptosis | apoptosis | melanoma | 34.44 | NEG_AFFECTS | 31 |
| cytokine-cytokine receptor interaction | cytokine | cytokinesis of the fertilized ovum | melanoma | 19.76 | PROCESS_OF | 6 |
| cytokine-cytokine receptor interaction | cytokine | cytokinesis of the fertilized ovum | melanoma | 19.76 | PROCESS_OF | 6 |
| micrornas in cancer | micrornas | micrornas | melanoma | 15.94 | DISRUPTS | 10 |
| lung fibrosis | lung | secondary malignant neoplasm of lung | melanoma | 15.86 | COEXISTS_WITH | 7 |
| mir-509-3p alteration of yap1/ecm axis | extracellular matrix | melanoma cell | extracellular matrix | 15.27 | PART_OF | 10 |
| mir-509-3p alteration of yap1/ecm axis | extracellular matrix | melanoma cell | extracellular matrix | 15.27 | PART_OF | 10 |
| c-type lectin receptor signaling pathway | lectin | galectin 1 | melanoma cell | 14.41 | PART_OF | 3 |
| response to elevated platelet cytosolic ca2+ | platelet | platelet activating factor receptor | melanoma | 14.07 | ASSOCIATED_WITH | 5 |
| tnf signaling pathway | tnf | tnfrsf10b gene\|tnfrsf10b | melanoma cell | 12.74 | INTERACTS_WITH | 4 |
| tnf signaling pathway | tnf | tnfrsf19 | melanoma | 12.36 | PREDISPOSES | 2 |
| lung fibrosis | lung | melanoma | secondary malignant neoplasm of lung | 11.90 | COEXISTS_WITH | 4 |
| apoptosis-related network due to altered notch3 in ovarian cancer | apoptosis | apoptosis inhibiting proteins | melanoma | 11.35 | ASSOCIATED_WITH | 6 |
| assembly of collagen fibrils and other multimeric structures | collagen | collagen type i | melanoma cell | 11.16 | INTERACTS_WITH | 4 |
| lung fibrosis | lung | melanoma | carcinoma, non-small-cell lung | 11.04 | PRECEDES | 2 |
| tnf signaling pathway | tnf | tnfsf6 gene\|faslg | melanoma | 10.86 | NEG_PREDISPOSES | 2 |
| assembly of collagen fibrils and other multimeric structures | collagen | collagenase 3 | melanoma | 10.85 | AFFECTS | 3 |
| tnf signaling pathway | tumor necrosis factor | melanoma cell | tumor necrosis factor ligand superfamily member 6\|faslg | 10.83 | LOCATION_OF | 7 |
| apoptosis-related network due to altered notch3 in ovarian cancer | apoptosis | anti-apoptosis | melanoma | 10.80 | PROCESS_OF | 3 |
| photodynamic therapy-induced ap-1 survival signaling. | ap-1 | melanoma cell | transcription factor ap-1\|fos\|fosb\|jun\|junb\|jund | 10.67 | LOCATION_OF | 3 |
| photodynamic therapy-induced nf-kb survival signaling | nf-kappab | receptor activator of nf-kappab | melanoma cell | 10.63 | INTERACTS_WITH | 2 |
| response to elevated platelet cytosolic ca2+ | ca2+ | oca2 | melanoma | 10.57 | PREDISPOSES | 2 |
| response to elevated platelet cytosolic ca2+ | platelet | melanoma cell | platelet activating factor receptor | 10.52 | PRODUCES | 3 |
| response to elevated platelet cytosolic ca2+ | platelet | melanoma | platelet-derived growth factor receptor | 9.73 | NEG_PRODUCES | 2 |
| apoptosis-related network due to altered notch3 in ovarian cancer | apoptosis | melanoma cell | apoptosis | 9.71 | NEG_LOCATION_OF | 6 |
| c-type lectin receptor signaling pathway | lectin | galectin 3 | melanoma cell | 9.13 | NEG_ADMINISTERED_TO | 1 |
| assembly of collagen fibrils and other multimeric structures | collagen | melanoma | interstitial collagenase | 8.93 | PRODUCES | 4 |
| transcriptional misregulation in cancer | transcriptional | melanoma cell | transcriptional regulation | 8.37 | LOCATION_OF | 4 |
| tnf signaling pathway | tumor necrosis factor | melanoma cell | tumor necrosis factor ligand superfamily member 6\|faslg | 8.26 | PRODUCES | 5 |
| malaria | malaria | melanoma cell | aminoquinoline antimalarial | 8.20 | LOCATION_OF | 1 |
| inflammatory response pathway | inflammatory | murine macrophage inflammatory protein 2 | melanoma | 7.94 | AUGMENTS | 1 |
| tnf signaling pathway | tumor necrosis factor | tumor necrosis factors | melanoma cell | 7.84 | INTERACTS_WITH | 4 |
| tnf signaling pathway | tnf | tnfrsf19 | melanoma cell | 7.80 | PART_OF | 1 |
| lung fibrosis | lung | secondary malignant neoplasm of lung | melanoma | 7.79 | CAUSES | 2 |
| apoptosis-related network due to altered notch3 in ovarian cancer | apoptosis | apoptosis | melanoma | 7.52 | AFFECTS | 20 |
| tnf signaling pathway | tumor necrosis factor | tumor necrosis factor ligand superfamily member 6 | melanoma cell | 7.49 | ADMINISTERED_TO | 1 |
| micrornas in cancer | micrornas | micrornas | melanoma cell | 7.46 | ADMINISTERED_TO | 3 |
| tnf signaling pathway | tnf | melanoma cell | tnf receptor-associated factor 2 | 7.40 | LOCATION_OF | 2 |
| micrornas in cancer | micrornas | micrornas | melanoma | 7.38 | CAUSES | 4 |
| apoptosis-related network due to altered notch3 in ovarian cancer | apoptosis | x-linked inhibitor of apoptosis protein | melanoma | 7.35 | TREATS | 2 |
| tnf signaling pathway | tnf | melanoma cell | tnf protein, human\|tnf | 7.33 | INTERACTS_WITH | 5 |
| tnf signaling pathway | tnf | tnfsf6 gene\|faslg | melanoma cell | 7.20 | ADMINISTERED_TO | 1 |
| tnf signaling pathway | tnf | melanoma cell | tnfsf6 gene\|faslg | 7.12 | LOCATION_OF | 2 |
| interleukin-6 family signaling | interleukin-6 | melanoma cell | interleukin-6 | 7.06 | INTERACTS_WITH | 4 |
| cytokine-cytokine receptor interaction | cytokine | recombinant cytokines | melanoma | 7.02 | NEG_AFFECTS | 1 |
| cytokine-cytokine receptor interaction | cytokine | recombinant cytokines | melanoma | 7.02 | NEG_AFFECTS | 1 |
| tnf signaling pathway | tumor necrosis factor | melanoma cell | tumor necrosis factor ligand superfamily member 6\|fas | 6.85 | LOCATION_OF | 3 |
| tnf signaling pathway | tnf | tnf protein, human\|tnf | melanoma cell | 6.76 | PART_OF | 4 |
| tnf signaling pathway | tumor necrosis factor | tumor necrosis factor ligand superfamily member 6\|faslg | melanoma cell | 6.67 | INTERACTS_WITH | 3 |
| tnf signaling pathway | tumor necrosis factor | melanoma cell | tumor necrosis factor receptor | 6.57 | LOCATION_OF | 3 |
| tnf signaling pathway | tnf | efna1\|tnfaip1 | melanoma | 6.47 | AFFECTS | 1 |
| assembly of collagen fibrils and other multimeric structures | collagen | melanoma cell | collagen type iv | 6.31 | INTERACTS_WITH | 2 |
| tnf signaling pathway | tnf | tnf gene\|tnf | melanoma cell | 6.30 | PART_OF | 2 |
| tnf signaling pathway | tnf | tnfrsf10c | melanoma | 6.25 | AFFECTS | 1 |
| tnf signaling pathway | tnf | hla-dr4 antigen\|hla-drb4\|tnfrsf10a | melanoma | 6.19 | PART_OF | 1 |
| assembly of collagen fibrils and other multimeric structures | collagen | neutrophil collagenase | melanoma | 6.15 | ASSOCIATED_WITH | 5 |
| c-type lectin receptor signaling pathway | lectin | galectin 1 | melanoma cell | 6.03 | NEG_INTERACTS_WITH | 1 |
| apoptosis-related network due to altered notch3 in ovarian cancer | apoptosis | x-linked inhibitor of apoptosis protein | melanoma cell | 5.93 | AUGMENTS | 1 |
| senescence and autophagy in cancer | autophagy | autophagy | melanoma cell | 5.90 | AFFECTS | 4 |
| lung fibrosis | lung | melanoma | lung | 5.74 | AFFECTS | 6 |
| assembly of collagen fibrils and other multimeric structures | collagen | collagen type iv | melanoma cell | 5.74 | INTERACTS_WITH | 2 |
| apoptosis-related network due to altered notch3 in ovarian cancer | apoptosis | melanoma cell | anti-apoptosis | 5.73 | NEG_LOCATION_OF | 1 |
| prostaglandin synthesis and regulation | prostaglandin | melanoma cell | prostaglandin e receptor | 5.69 | INTERACTS_WITH | 1 |
| tnf signaling pathway | tnf | tnfrsf4 | melanoma | 5.66 | PREVENTS | 1 |
| assembly of collagen fibrils and other multimeric structures | collagen | interstitial collagenase | melanoma cell | 5.65 | ADMINISTERED_TO | 1 |
| tnf signaling pathway | tumor necrosis factor | melanoma cell | tumor necrosis factor ligand superfamily member 6\|faslg | 5.59 | INTERACTS_WITH | 2 |
| c-type lectin receptor signaling pathway | lectin | galectin 1 | melanoma cell | 5.59 | INTERACTS_WITH | 2 |
| tnf signaling pathway | tnf | melanoma cell | tnfrsf10b gene\|tnfrsf10b | 5.55 | INTERACTS_WITH | 2 |
| bladder cancer | bladder | bladder | melanomma | 5.55 | LOCATION_OF | 1 |
| micrornas in cancer | micrornas | micrornas | melanoma | 5.53 | AFFECTS | 18 |
| tnf signaling pathway | tumor necrosis factor | melanoma | tumor necrosis factor ligand superfamily member 6\|fas | 5.47 | PRODUCES | 2 |
| il-17 signaling pathway | interleukin-17 | interleukin-17 receptor | melanoma | 5.37 | AFFECTS | 1 |
| tnf signaling pathway | tnf | melanoma cell | tnfrsf6 gene\|fas | 5.36 | NEG_INTERACTS_WITH | 1 |
| tnf signaling pathway | tumor necrosis factor | tumor necrosis factor receptor superfamily, member 10b | melanoma cell | 5.31 | AFFECTS | 1 |
| tnf signaling pathway | tumor necrosis factor | melanoma cell | tumor necrosis factor receptor | 5.30 | PRODUCES | 2 |
| response to elevated platelet cytosolic ca2+ | platelet | platelet-derived growth factor beta receptor | melanoma | 5.14 | NEG_ASSOCIATED_WITH | 1 |
| tnf signaling pathway | tnf | tnfrsf10b gene\|tnfrsf10b | melanoma cell | 5.13 | ADMINISTERED_TO | 1 |
| tnf signaling pathway | tumor necrosis factor | tumor necrosis factor-beta | melanoma | 5.09 | NEG_TREATS | 1 |
| tnf signaling pathway | tnf | tnfsf10 gene\|tnfsf10 | melanoma | 5.09 | ASSOCIATED_WITH | 3 |
| response to elevated platelet cytosolic ca2+ | platelet | melanoma cell | platelet-derived growth factor receptor | 5.05 | PRODUCES | 2 |
| photodynamic therapy-induced nf-kb survival signaling | nf-kappab | receptor activator of nf-kappab | melanoma cell | 5.02 | STIMULATES | 1 |
| tnf signaling pathway | tnf | tnfrsf19 | melanoma cell | 4.96 | INTERACTS_WITH | 1 |
| tnf signaling pathway | tnf | tnfrsf12a | melanoma cell | 4.92 | STIMULATES | 1 |
| assembly of collagen fibrils and other multimeric structures | collagen | melanoma cell | collagenase | 4.90 | LOCATION_OF | 3 |
| tnf signaling pathway | tumor necrosis factor | tumor necrosis factor ligand superfamily member 6\|fas | melanoma | 4.88 | NEG_PART_OF | 1 |
| cytokine-cytokine receptor interaction | cytokine | melanoma cell | cytokinesis of the fertilized ovum | 4.81 | LOCATION_OF | 4 |
| cytokine-cytokine receptor interaction | cytokine | melanoma cell | cytokinesis of the fertilized ovum | 4.81 | LOCATION_OF | 4 |
| tnf signaling pathway | tumor necrosis factor | tumor necrosis factor ligand superfamily member 6 | melanoma | 4.80 | PREDISPOSES | 1 |
| response to elevated platelet cytosolic ca2+ | ca2+ | brca2 gene\|brca2 | melanoma | 4.77 | PREDISPOSES | 3 |
| assembly of collagen fibrils and other multimeric structures | collagen | neutrophil collagenase | melanoma | 4.76 | DISRUPTS | 1 |
| apoptosis-related network due to altered notch3 in ovarian cancer | apoptosis | apoptosis regulator | melanoma cell | 4.75 | INTERACTS_WITH | 1 |
| interleukin-6 family signaling | interleukin-6 | interleukin-6 | melanoma cell | 4.72 | PART_OF | 2 |
| apoptosis-related network due to altered notch3 in ovarian cancer | apoptosis | apoptosis | melanoma | 4.68 | NEG_PROCESS_OF | 1 |
| apoptosis-related network due to altered notch3 in ovarian cancer | apoptosis | tnf-related apoptosis-inducing ligand | melanoma | 4.62 | ASSOCIATED_WITH | 2 |
| tnf signaling pathway | tnf | tnf-related apoptosis-inducing ligand | melanoma | 4.62 | ASSOCIATED_WITH | 2 |
| micrornas in cancer | micrornas | micrornas | melanoma cell | 4.57 | PART_OF | 3 |
| assembly of collagen fibrils and other multimeric structures | collagen | melanoma cell | fibrillar collagen | 4.56 | INTERACTS_WITH | 1 |
| tnf signaling pathway | tnf | tnfsf10 gene\|tnfsf10 | melanoma cell | 4.55 | DISRUPTS | 1 |
| prostaglandin synthesis and regulation | prostaglandin | prostaglandin a1 | melanoma cell | 4.52 | DISRUPTS | 1 |
| tnf signaling pathway | tnf | tnfrsf10b gene\|tnfrsf10b | melanoma | 4.51 | PREVENTS | 1 |
| c-type lectin receptor signaling pathway | lectin | galectin 1 | melanoma | 4.46 | PREVENTS | 1 |
| tnf signaling pathway | tnf | melanoma | tnfsf6 gene\|faslg | 4.43 | PRODUCES | 1 |
| response to elevated platelet cytosolic ca2+ | platelet | platelet factor 4 | melanoma | 4.43 | DISRUPTS | 1 |
| tnf signaling pathway | tumor necrosis factor | tumor necrosis factor ligand superfamily member 6\|fas | melanoma | 4.42 | ASSOCIATED_WITH | 4 |
| tnf signaling pathway | tumor necrosis factor | melanoma | tumor necrosis factor receptor 11b | 4.34 | PRODUCES | 2 |
| lung fibrosis | lung | melanoma cell | carcinoma, non-small-cell lung | 4.33 | LOCATION_OF | 2 |
| prostaglandin synthesis and regulation | prostaglandin | melanoma cell | prostaglandin a2 | 4.32 | LOCATION_OF | 1 |
| assembly of collagen fibrils and other multimeric structures | collagen | melanoma cell | procollagen-proline dioxygenase | 4.31 | INTERACTS_WITH | 1 |
| cytokine-cytokine receptor interaction | cytokine | cytokine gene | melanoma cell | 4.28 | ADMINISTERED_TO | 1 |
| cytokine-cytokine receptor interaction | cytokine | cytokine gene | melanoma cell | 4.28 | ADMINISTERED_TO | 1 |
| c-type lectin receptor signaling pathway | lectin | galectin 1 | melanoma | 4.26 | ASSOCIATED_WITH | 5 |
| tnf signaling pathway | tumor necrosis factor | tumor necrosis factor receptor superfamily, member 10b | melanoma cell | 4.22 | INTERACTS_WITH | 1 |
| photodynamic therapy-induced nf-kb survival signaling | nf-kappab | nf-kappab-inducing kinase | melanoma cell | 4.15 | STIMULATES | 1 |
| lung fibrosis | lung | secondary malignant neoplasm of lung | melanoma | 4.12 | MANIFESTATION_OF | 1 |
| micrornas in cancer | micrornas | micrornas | melanoma | 4.11 | ASSOCIATED_WITH | 97 |
| tnf signaling pathway | tnf | tnfrsf10b gene\|tnfrsf10b | melanoma cell | 4.07 | NEG_AFFECTS | 1 |
| tnf signaling pathway | tnf | cux1\|hcls1\|tnfrsf1b\|psip1\|siglec7 | melanoma cell | 4.07 | AFFECTS | 1 |
| tnf signaling pathway | tumor necrosis factor | tumor necrosis factor receptor superfamily, member 10b | melanoma | 4.05 | ASSOCIATED_WITH | 2 |
| apoptosis-related network due to altered notch3 in ovarian cancer | apoptosis | melanoma cell | anti-apoptosis | 4.03 | LOCATION_OF | 2 |
| apoptosis-related network due to altered notch3 in ovarian cancer | apoptosis | apoptosis inhibiting proteins | melanoma | 4.01 | AUGMENTS | 1 |
| apoptosis-related network due to altered notch3 in ovarian cancer | apoptosis | apoptosis inhibiting proteins | melanoma | 4.00 | PREDISPOSES | 1 |
| senescence and autophagy in cancer | autophagy | autophagy | melanoma | 3.99 | AFFECTS | 7 |
| micrornas in cancer | micrornas | micrornas | melanoma | 3.97 | NEG_ASSOCIATED_WITH | 3 |
| photodynamic therapy-induced nf-kb survival signaling | nf-kappab | nf-kappab-inducing kinase | melanoma cell | 3.97 | INTERACTS_WITH | 1 |
| tnf signaling pathway | tnf | tnfrsf10a gene\|tnfrsf10a | melanoma cell | 3.96 | INTERACTS_WITH | 1 |
| tnf signaling pathway | tumor necrosis factor | melanoma cell | tumor necrosis factor ligand superfamily member 6 | 3.95 | PRODUCES | 1 |
| class a/1 (rhodopsin-like receptors) | rhodopsin | rhodopsin kinase\|rho | melanoma | 3.95 | ASSOCIATED_WITH | 1 |
| apoptosis-related network due to altered notch3 in ovarian cancer | apoptosis | melanoma cell | apoptosis regulatory proteins | 3.93 | LOCATION_OF | 1 |
| tnf signaling pathway | tnf | melanoma cell | tnfsf6 gene\|faslg | 3.91 | PRODUCES | 1 |
| prostaglandin synthesis and regulation | prostaglandin | prostaglandin a1 | melanoma | 3.90 | AFFECTS | 1 |
| tnf signaling pathway | tumor necrosis factor | tumor necrosis factor ligand superfamily member 6\|fas | melanoma | 3.88 | AUGMENTS | 1 |
| tnf signaling pathway | tumor necrosis factor | melanoma cell | tumor necrosis factor ligand superfamily member 6\|faslg | 3.82 | NEG_LOCATION_OF | 1 |
| apoptosis-related network due to altered notch3 in ovarian cancer | apoptosis | apoptosis inhibiting proteins | melanoma cell | 3.82 | DISRUPTS | 1 |
| tnf signaling pathway | tnf | tnf gene\|tnf | melanoma | 3.82 | TREATS | 2 |
| tnf signaling pathway | tnf | tnf gene\|tnf | melanoma cell | 3.81 | INTERACTS_WITH | 2 |
| il-18 signaling pathway | interleukin-18 | melanoma cell | interleukin-18 receptor | 3.80 | LOCATION_OF | 1 |
| cytokine-cytokine receptor interaction | cytokine | cytokine inducible sh2-containing protein\|cish | melanoma | 3.80 | PREDISPOSES | 1 |
| cytokine-cytokine receptor interaction | cytokine | cytokine inducible sh2-containing protein\|cish | melanoma | 3.80 | PREDISPOSES | 1 |
| assembly of collagen fibrils and other multimeric structures | collagen | melanoma cell | collagenase 3 | 3.79 | LOCATION_OF | 2 |
| c-type lectin receptor signaling pathway | lectin | galectin 3 | melanoma | 3.75 | AFFECTS | 2 |
| prostaglandin synthesis and regulation | prostaglandin | melanoma cell | 9-deoxy-delta-9-prostaglandin d2 | 3.72 | LOCATION_OF | 1 |
| response to elevated platelet cytosolic ca2+ | platelet | platelet activating factor receptor | melanoma cell | 3.71 | INTERACTS_WITH | 1 |
| tnf signaling pathway | tumor necrosis factor | tumor necrosis factor ligand superfamily member 6 | melanoma | 3.70 | PART_OF | 1 |
| assembly of collagen fibrils and other multimeric structures | collagen | collagen type iv | melanoma cell | 3.64 | STIMULATES | 1 |
| bladder cancer | bladder | bladder | melanoma | 3.64 | LOCATION_OF | 92 |
| response to elevated platelet cytosolic ca2+ | platelet | platelet-derived growth factor beta receptor | melanoma | 3.63 | ASSOCIATED_WITH | 2 |
| tnf signaling pathway | tumor necrosis factor | melanoma | tumor necrosis factor-alpha | 3.61 | PRODUCES | 1 |
| tnf signaling pathway | tumor necrosis factor | tumor necrosis factors\|tnf | melanoma | 3.61 | ASSOCIATED_WITH | 2 |
| tnf signaling pathway | tnf | tumor necrosis factors\|tnf | melanoma | 3.61 | ASSOCIATED_WITH | 2 |
| apoptosis-related network due to altered notch3 in ovarian cancer | apoptosis | tnf-related apoptosis-inducing ligand\|tnf | melanoma | 3.61 | DISRUPTS | 1 |
| tnf signaling pathway | tnf | tnf-related apoptosis-inducing ligand\|tnf | melanoma | 3.61 | DISRUPTS | 1 |
| c-type lectin receptor signaling pathway | lectin | galectin 3 | melanoma | 3.58 | PREVENTS | 1 |
| tnf signaling pathway | tnf | tnf | melanoma cell | 3.55 | AUGMENTS | 1 |
| tnf signaling pathway | tumor necrosis factor | tumor necrosis factors | melanoma | 3.54 | PART_OF | 2 |
| tnf signaling pathway | tnf | cux1\|hcls1\|tnfrsf1b\|psip1\|siglec7 | melanoma | 3.51 | PART_OF | 1 |
| cytokine-cytokine receptor interaction | cytokine | melanoma cell | cytokine inducible sh2-containing protein\|cish | 3.49 | PRODUCES | 1 |
| cytokine-cytokine receptor interaction | cytokine | melanoma cell | cytokine inducible sh2-containing protein\|cish | 3.49 | PRODUCES | 1 |
| tnf signaling pathway | tnf | tnfrsf19 | melanoma | 3.49 | ASSOCIATED_WITH | 1 |
| tnf signaling pathway | tumor necrosis factor | tumor necrosis factor receptor | melanoma | 3.41 | CAUSES | 1 |
| tnf signaling pathway | tnf | tnfrsf10b gene\|tnfrsf10b | melanoma cell | 3.38 | AFFECTS | 1 |
| assembly of collagen fibrils and other multimeric structures | collagen | collagen type vi | melanoma | 3.37 | ASSOCIATED_WITH | 2 |
| bladder cancer | bladder | melanoma | bladder | 3.35 | AFFECTS | 2 |
| response to elevated platelet cytosolic ca2+ | platelet | platelet aggregation | melanoma | 3.35 | PROCESS_OF | 2 |
| interleukin-6 family signaling | interleukin-6 | melanoma | interleukin-6\|il6 | 3.34 | NEG_PRODUCES | 1 |
| cytokine-cytokine receptor interaction | cytokine | melanoma cell | cytokine | 3.30 | INTERACTS_WITH | 4 |
| cytokine-cytokine receptor interaction | cytokine | melanoma cell | cytokine | 3.30 | INTERACTS_WITH | 4 |
| response to elevated platelet cytosolic ca2+ | platelet | platelet factor 4\|pf4 | melanoma | 3.30 | DISRUPTS | 1 |
| mir-509-3p alteration of yap1/ecm axis | extracellular matrix | extracellular matrix proteins | melanoma | 3.29 | DISRUPTS | 1 |
| mir-509-3p alteration of yap1/ecm axis | extracellular matrix | extracellular matrix proteins | melanoma | 3.29 | DISRUPTS | 1 |
| response to elevated platelet cytosolic ca2+ | platelet | melanoma cell | platelet-derived growth factor alpha receptor | 3.25 | LOCATION_OF | 1 |
| assembly of collagen fibrils and other multimeric structures | collagen | collagenase | melanoma cell | 3.23 | ADMINISTERED_TO | 1 |
| micrornas in cancer | micrornas | micrornas | melanoma cell | 3.22 | AUGMENTS | 2 |
| apoptosis-related network due to altered notch3 in ovarian cancer | apoptosis | anti-apoptosis | melanoma | 3.21 | NEG_AFFECTS | 1 |
| transcriptional misregulation in cancer | transcriptional | melanoma cell | transcriptional activation | 3.18 | AFFECTS | 1 |
| tnf signaling pathway | tnf | melanoma cell | tnfrsf5 gene\|cd40 | 3.17 | LOCATION_OF | 3 |
| il-18 signaling pathway | interleukin-18 | melanoma cell | interleukin-18 | 3.15 | INTERACTS_WITH | 1 |
| tnf signaling pathway | tnf | tnfrsf9 | melanoma | 3.11 | ASSOCIATED_WITH | 2 |
| tnf signaling pathway | tnf | tnf gene\|tnf | melanoma | 3.11 | AFFECTS | 2 |
| response to elevated platelet cytosolic ca2+ | platelet | platelet aggregation | melanoma cell | 3.10 | AFFECTS | 1 |
| tnf signaling pathway | tnf | tnfsf9 | melanoma | 3.10 | ASSOCIATED_WITH | 1 |
| assembly of collagen fibrils and other multimeric structures | collagen | collagenase | melanoma | 3.09 | DISRUPTS | 1 |
| lung fibrosis | lung | carcinoma, non-small-cell lung | melanoma | 3.09 | COEXISTS_WITH | 2 |
| lung fibrosis | lung | melanoma | carcinoma, non-small-cell lung | 3.07 | COEXISTS_WITH | 3 |
| c-type lectin receptor signaling pathway | lectin | galectin 3 | melanoma | 3.05 | ASSOCIATED_WITH | 9 |
| tnf signaling pathway | tumor necrosis factor | melanoma cell | tumor necrosis factor receptor superfamily, member 10b | 3.05 | LOCATION_OF | 1 |
| tnf signaling pathway | tumor necrosis factor | tumor necrosis factors | melanoma cell | 3.03 | STIMULATES | 2 |
| apoptosis-related network due to altered notch3 in ovarian cancer | apoptosis | casp8 and fadd-like apoptosis regulating protein\|casp8 | melanoma | 3.02 | ASSOCIATED_WITH | 1 |
| cytokine-cytokine receptor interaction | cytokine | melanoma cell | cytokine gene | 3.02 | LOCATION_OF | 2 |
| cytokine-cytokine receptor interaction | cytokine | melanoma cell | cytokine gene | 3.02 | LOCATION_OF | 2 |
| cytokine-cytokine receptor interaction | cytokine | cytokine | melanoma | 3.02 | PART_OF | 5 |
| cytokine-cytokine receptor interaction | cytokine | cytokine | melanoma | 3.02 | PART_OF | 5 |
| il-17 signaling pathway | interleukin-17 | interleukin-17 | melanoma | 2.99 | PART_OF | 1 |
| tnf signaling pathway | tumor necrosis factor | tumor necrosis factors | melanoma cell | 2.97 | NEG_AFFECTS | 1 |
| interleukin-6 family signaling | interleukin-6 | melanoma cell | interleukin-6\|il6 | 2.95 | INTERACTS_WITH | 1 |
| cytokine-cytokine receptor interaction | cytokine | melanoma cell | cytokine gene | 2.94 | INTERACTS_WITH | 1 |
| cytokine-cytokine receptor interaction | cytokine | melanoma cell | cytokine gene | 2.94 | INTERACTS_WITH | 1 |
| tnf signaling pathway | tnf | tnfrsf6 gene\|fas | melanoma cell | 2.91 | INTERACTS_WITH | 1 |
| tnf signaling pathway | tumor necrosis factor | tumor necrosis factor receptor superfamily, member 10b\|tnfrsf10b | melanoma | 2.91 | ASSOCIATED_WITH | 2 |
| tnf signaling pathway | tnf | tumor necrosis factor receptor superfamily, member 10b\|tnfrsf10b | melanoma | 2.91 | ASSOCIATED_WITH | 2 |
| tnf signaling pathway | tnf | ndufb3\|tnfaip1 | melanoma | 2.89 | NEG_ASSOCIATED_WITH | 1 |
| tnf signaling pathway | tnf | melanoma cell | tnfrsf5 gene\|cd40 | 2.89 | PRODUCES | 2 |
| cytokine-cytokine receptor interaction | cytokine | melanoma cell | small inducible cytokine a21\|ccl21 | 2.87 | PRODUCES | 1 |
| response to elevated platelet cytosolic ca2+ | platelet | melanoma cell | platelet-derived growth factor bb | 2.87 | PRODUCES | 1 |
| cytokine-cytokine receptor interaction | cytokine | melanoma cell | small inducible cytokine a21\|ccl21 | 2.87 | PRODUCES | 1 |
| tnf signaling pathway | tnf | tnfrsf10b gene\|tnfrsf10b | melanoma | 2.86 | NEG_AFFECTS | 1 |
| lung fibrosis | lung | secondary malignant neoplasm of lung | melanoma | 2.77 | AFFECTS | 1 |
| apoptosis-related network due to altered notch3 in ovarian cancer | apoptosis | melanoma | apoptosis | 2.74 | NEG_AFFECTS | 3 |
| response to elevated platelet cytosolic ca2+ | platelet | melanoma | platelet-derived growth factor receptor | 2.71 | PRODUCES | 1 |
| assembly of collagen fibrils and other multimeric structures | collagen | collagen type iv | melanoma | 2.71 | DISRUPTS | 1 |
| interleukin-6 family signaling | interleukin-6 | interleukin-6 | melanoma cell | 2.67 | NEG_AFFECTS | 1 |
| tnf signaling pathway | tumor necrosis factor | tumor necrosis factor ligand superfamily member 6 | melanoma | 2.67 | ASSOCIATED_WITH | 1 |
| apoptosis-related network due to altered notch3 in ovarian cancer | apoptosis | melanoma cell | tnf-related apoptosis-inducing ligand\|tnf | 2.66 | LOCATION_OF | 1 |
| tnf signaling pathway | tnf | melanoma cell | tnf-related apoptosis-inducing ligand\|tnf | 2.66 | LOCATION_OF | 1 |
| assembly of collagen fibrils and other multimeric structures | collagen | collagen type i | melanoma cell | 2.65 | PART_OF | 1 |
| prostaglandin synthesis and regulation | prostaglandin | prostaglandin-endoperoxide synthase 2 (prostaglandin g/h synthase and cyclooxygenase), human | melanoma | 2.64 | DISRUPTS | 1 |
| prostaglandin synthesis and regulation | prostaglandin | 9-deoxy-delta-9-prostaglandin d2 | melanoma | 2.57 | ASSOCIATED_WITH | 1 |
| pertussis | pertussis | pertussis toxin | melanoma cell | 2.57 | PART_OF | 1 |
| cytokine-cytokine receptor interaction | cytokine | cytokine | melanoma cell | 2.55 | INTERACTS_WITH | 5 |
| cytokine-cytokine receptor interaction | cytokine | cytokine | melanoma cell | 2.55 | INTERACTS_WITH | 5 |
| apoptosis-related network due to altered notch3 in ovarian cancer | apoptosis | anti-apoptosis | melanoma cell | 2.54 | AFFECTS | 1 |
| apoptosis-related network due to altered notch3 in ovarian cancer | apoptosis | tnf-related apoptosis-inducing ligand\|tnf | melanoma | 2.54 | ASSOCIATED_WITH | 2 |
| tnf signaling pathway | tnf | tnf-related apoptosis-inducing ligand\|tnf | melanoma | 2.54 | ASSOCIATED_WITH | 2 |
| tnf signaling pathway | tnf | tnf protein, human\|tnf | melanoma | 2.53 | TREATS | 3 |
| tnf signaling pathway | tnf | tnfrsf10b gene\|tnfrsf10b | melanoma | 2.52 | ASSOCIATED_WITH | 4 |
| tnf signaling pathway | tumor necrosis factor | tumor necrosis factor receptor | melanoma | 2.52 | ASSOCIATED_WITH | 3 |
| tnf signaling pathway | tumor necrosis factor | tumor necrosis factor ligand superfamily member 6\|faslg | melanoma | 2.50 | ASSOCIATED_WITH | 6 |
| tnf signaling pathway | tumor necrosis factor | melanoma cell | tumor necrosis factor receptor superfamily, member 10b\|tnfrsf10b | 2.46 | LOCATION_OF | 1 |
| tnf signaling pathway | tnf | melanoma cell | tumor necrosis factor receptor superfamily, member 10b\|tnfrsf10b | 2.46 | LOCATION_OF | 1 |
| tnf signaling pathway | tumor necrosis factor | tumor necrosis factor-alpha\|tnf | melanoma cell | 2.46 | STIMULATES | 2 |
| tnf signaling pathway | tnf | tumor necrosis factor-alpha\|tnf | melanoma cell | 2.46 | STIMULATES | 2 |
| il-18 signaling pathway | interleukin-18 | interleukin-18 | melanoma | 2.44 | PREDISPOSES | 2 |
| assembly of collagen fibrils and other multimeric structures | collagen | melanoma cell | interstitial collagenase | 2.44 | LOCATION_OF | 2 |
| apoptosis-related network due to altered notch3 in ovarian cancer | apoptosis | melanoma | anti-apoptosis | 2.42 | MANIFESTATION_OF | 1 |
| tnf signaling pathway | tnf | tnfrsf10a gene\|tnfrsf10a | melanoma | 2.41 | ASSOCIATED_WITH | 4 |
| assembly of collagen fibrils and other multimeric structures | collagen | collagenase | melanoma cell | 2.41 | INTERACTS_WITH | 1 |
| assembly of collagen fibrils and other multimeric structures | collagen | collagen type iv | melanoma | 2.40 | AFFECTS | 1 |
| c-type lectin receptor signaling pathway | lectin | melanoma | galectin 3 | 2.40 | PRODUCES | 1 |
| mir-509-3p alteration of yap1/ecm axis | extracellular matrix | melanoma cell | extracellular matrix proteins | 2.37 | INTERACTS_WITH | 1 |
| mir-509-3p alteration of yap1/ecm axis | extracellular matrix | melanoma cell | extracellular matrix proteins | 2.37 | INTERACTS_WITH | 1 |
| assembly of collagen fibrils and other multimeric structures | collagen | interstitial collagenase | melanoma | 2.36 | AFFECTS | 1 |
| tnf signaling pathway | tumor necrosis factor | recombinant tumor necrosis factor-alpha | melanoma cell | 2.36 | PART_OF | 1 |
| cytokine-cytokine receptor interaction | cytokine | cytokine | melanoma cell | 2.34 | NEG_AFFECTS | 1 |
| cytokine-cytokine receptor interaction | cytokine | cytokine | melanoma cell | 2.34 | NEG_AFFECTS | 1 |
| tnf signaling pathway | tnf | tnfrsf12a | melanoma | 2.31 | ASSOCIATED_WITH | 2 |
| tnf signaling pathway | tnf | tnfrsf10b gene\|tnfrsf10b | melanoma cell | 2.29 | PART_OF | 1 |
| photodynamic therapy-induced ap-1 survival signaling. | ap-1 | transcription factor ap-1 | melanoma | 2.29 | ASSOCIATED_WITH | 2 |
| tnf signaling pathway | tnf | tnf receptor-associated factor 2 | melanoma | 2.28 | ASSOCIATED_WITH | 1 |
| apoptosis-related network due to altered notch3 in ovarian cancer | apoptosis | apoptosis | melanoma | 2.28 | COEXISTS_WITH | 6 |
| response to elevated platelet cytosolic ca2+ | ca2+ | brca2 gene\|brca2 | melanoma | 2.28 | PART_OF | 1 |
| il-18 signaling pathway | interleukin-18 | interleukin-18 | melanoma cell | 2.28 | INTERACTS_WITH | 1 |
| lung fibrosis | lung | melanoma | secondary malignant neoplasm of lung | 2.25 | AFFECTS | 1 |
| tnf signaling pathway | tumor necrosis factor | tumor necrosis factor ligand superfamily member 6\|faslg | melanoma | 2.22 | DISRUPTS | 1 |
| tnf signaling pathway | tumor necrosis factor | tumor necrosis factor-alpha inhibitor | melanoma | 2.20 | ASSOCIATED_WITH | 2 |
| c-type lectin receptor signaling pathway | lectin | galectin 3 | melanoma cell | 2.18 | INTERACTS_WITH | 1 |
| tnf signaling pathway | tnf | tnf protein, human\|tnf | melanoma cell | 2.17 | NEG_AFFECTS | 1 |
| tnf signaling pathway | tumor necrosis factor | tumor necrosis factor ligand superfamily member 6\|fas | melanoma | 2.13 | AFFECTS | 1 |
| tnf signaling pathway | tnf | tnf protein, human\|tnf | melanoma cell | 2.12 | ADMINISTERED_TO | 1 |
| response to elevated platelet cytosolic ca2+ | platelet | melanoma cell | platelet activating factor | 2.10 | LOCATION_OF | 2 |
| mir-509-3p alteration of yap1/ecm axis | extracellular matrix | melanoma | extracellular matrix | 2.09 | PRODUCES | 1 |
| mir-509-3p alteration of yap1/ecm axis | extracellular matrix | melanoma | extracellular matrix | 2.09 | PRODUCES | 1 |
| interleukin-6 family signaling | interleukin-6 | interleukin-6 | melanoma | 2.07 | PREVENTS | 1 |
| assembly of collagen fibrils and other multimeric structures | collagen | collagen antibody | melanoma | 2.07 | ASSOCIATED_WITH | 1 |
| mir-509-3p alteration of yap1/ecm axis | extracellular matrix | extracellular matrix | melanoma | 2.06 | PART_OF | 3 |
| mir-509-3p alteration of yap1/ecm axis | extracellular matrix | extracellular matrix | melanoma | 2.06 | PART_OF | 3 |
| tnf signaling pathway | tumor necrosis factor | tumor necrosis factor ligand superfamily member 6\|faslg | melanoma | 2.05 | PREDISPOSES | 1 |
| tnf signaling pathway | tumor necrosis factor | melanoma | tumor necrosis factor-beta | 2.03 | PRODUCES | 1 |
| tnf signaling pathway | tumor necrosis factor | receptors, tumor necrosis factor, type ii | melanoma | 2.03 | ASSOCIATED_WITH | 1 |
| response to elevated platelet cytosolic ca2+ | platelet | platelet-derived growth factor | melanoma | 2.02 | PART_OF | 1 |
| interleukin-6 family signaling | interleukin-6 | melanoma cell | interleukin-6 | 2.01 | PRODUCES | 8 |
| tnf signaling pathway | tnf | tnf protein, human\|tnf | melanoma cell | 2.00 | INTERACTS_WITH | 3 |
| interleukin-6 family signaling | interleukin-6 | melanoma cell | interleukin-6\|il6 | 2.00 | PRODUCES | 2 |
| micrornas in cancer | micrornas | micrornas | melanoma | 1.99 | PREDISPOSES | 8 |
| tnf signaling pathway | tnf | hla-dr4 antigen\|hla-drb4\|tnfrsf10a | melanoma | 1.98 | ASSOCIATED_WITH | 1 |
| photodynamic therapy-induced ap-1 survival signaling. | ap-1 | melanoma cell | transcription factor ap-1 | 1.96 | LOCATION_OF | 1 |
| assembly of collagen fibrils and other multimeric structures | collagen | melanoma cell | collagen type i | 1.95 | LOCATION_OF | 2 |
| c-type lectin receptor signaling pathway | lectin | melanoma cell | galectin 3 | 1.94 | PRODUCES | 1 |
| tnf signaling pathway | tumor necrosis factor | melanoma | tumor necrosis factor-alpha\|tnf | 1.93 | NEG_PRODUCES | 1 |
| tnf signaling pathway | tnf | melanoma | tumor necrosis factor-alpha\|tnf | 1.93 | NEG_PRODUCES | 1 |
| tnf signaling pathway | tumor necrosis factor | tumor necrosis factor-alpha inhibitor\|tnf | melanoma | 1.91 | ASSOCIATED_WITH | 1 |
| tnf signaling pathway | tnf | tumor necrosis factor-alpha inhibitor\|tnf | melanoma | 1.91 | ASSOCIATED_WITH | 1 |
| tnf signaling pathway | tumor necrosis factor | tumor necrosis factors | melanoma | 1.90 | ASSOCIATED_WITH | 7 |
| cytokine-cytokine receptor interaction | cytokine | melanoma cell | cytokine inducible sh2-containing protein\|cish | 1.89 | LOCATION_OF | 1 |
| cytokine-cytokine receptor interaction | cytokine | melanoma cell | cytokine inducible sh2-containing protein\|cish | 1.89 | LOCATION_OF | 1 |
| tnf signaling pathway | tnf | melanoma cell | tnfrsf10a gene\|tnfrsf10a | 1.88 | LOCATION_OF | 1 |
| assembly of collagen fibrils and other multimeric structures | collagen | melanoma cell | collagenase 3 | 1.88 | PRODUCES | 1 |
| response to elevated platelet cytosolic ca2+ | platelet | platelet-derived growth factor beta receptor\|pdgfrb | melanoma | 1.82 | ASSOCIATED_WITH | 1 |
| senescence and autophagy in cancer | autophagy | autophagy | melanoma | 1.80 | COEXISTS_WITH | 2 |
| response to elevated platelet cytosolic ca2+ | platelet | platelet-derived growth factor | melanoma | 1.78 | ASSOCIATED_WITH | 4 |
| prostaglandin synthesis and regulation | prostaglandin | prostaglandins | melanoma | 1.76 | DISRUPTS | 1 |
| micrornas in cancer | micrornas | micrornas | melanoma cell | 1.76 | AFFECTS | 3 |
| cytokine-cytokine receptor interaction | cytokine | cytokine gene | melanoma | 1.75 | ASSOCIATED_WITH | 3 |
| cytokine-cytokine receptor interaction | cytokine | cytokine gene | melanoma | 1.75 | ASSOCIATED_WITH | 3 |
| tnf signaling pathway | tumor necrosis factor | tumor necrosis factor-alpha\|tnf | melanoma | 1.72 | DISRUPTS | 1 |
| tnf signaling pathway | tnf | tumor necrosis factor-alpha\|tnf | melanoma | 1.72 | DISRUPTS | 1 |
| apoptosis-related network due to altered notch3 in ovarian cancer | apoptosis | melanoma cell | apoptosis inducing factor | 1.69 | LOCATION_OF | 1 |
| c-type lectin receptor signaling pathway | lectin | melanoma cell | galectin 1 | 1.69 | LOCATION_OF | 1 |
| apoptosis-related network due to altered notch3 in ovarian cancer | apoptosis | melanoma | apoptosis | 1.69 | COEXISTS_WITH | 3 |
| transcriptional misregulation in cancer | transcriptional | melanoma | transcriptional activation | 1.69 | AFFECTS | 3 |
| interleukin-6 family signaling | interleukin-6 | interleukin-6 | melanoma cell | 1.68 | INTERACTS_WITH | 2 |
| micrornas in cancer | micrornas | micrornas | melanoma | 1.67 | NEG_PREDISPOSES | 1 |
| tnf signaling pathway | tnf | melanoma | tnfrsf5 gene\|cd40 | 1.66 | PRODUCES | 1 |
| assembly of collagen fibrils and other multimeric structures | collagen | melanoma cell | collagen type iv | 1.63 | PRODUCES | 1 |
| tnf signaling pathway | tumor necrosis factor | melanoma cell | tumor necrosis factor-beta | 1.61 | PRODUCES | 1 |
| tnf signaling pathway | tumor necrosis factor | tumor necrosis factors | melanoma | 1.60 | DISRUPTS | 1 |
| assembly of collagen fibrils and other multimeric structures | collagen | collagen type xvii | melanoma | 1.56 | ASSOCIATED_WITH | 1 |
| assembly of collagen fibrils and other multimeric structures | collagen | interstitial collagenase | melanoma | 1.56 | ASSOCIATED_WITH | 3 |
| tnf signaling pathway | tnf | tnf protein, human\|tnf | melanoma | 1.55 | AFFECTS | 3 |
| cytokine-cytokine receptor interaction | cytokine | cytokine | melanoma | 1.55 | TREATS | 1 |
| cytokine-cytokine receptor interaction | cytokine | cytokine | melanoma | 1.55 | TREATS | 1 |
| lung fibrosis | lung | carcinoma, non-small-cell lung | melanoma | 1.53 | AFFECTS | 1 |
| lung fibrosis | lung | lung | melanoma | 1.53 | NEG_LOCATION_OF | 2 |
| prostaglandin synthesis and regulation | prostaglandin | prostaglandin r2 d-isomerase | melanoma | 1.52 | ASSOCIATED_WITH | 1 |
| prostaglandin synthesis and regulation | prostaglandin | prostaglandin-endoperoxide synthase 2 (prostaglandin g/h synthase and cyclooxygenase), human | melanoma | 1.52 | ASSOCIATED_WITH | 3 |
| response to elevated platelet cytosolic ca2+ | platelet | melanoma | platelet activating factor | 1.51 | PRODUCES | 1 |
| assembly of collagen fibrils and other multimeric structures | collagen | interstitial collagenase\|mmp1 | melanoma | 1.50 | ASSOCIATED_WITH | 1 |
| interleukin-6 family signaling | interleukin-6 | interleukin-6 | melanoma | 1.50 | PREDISPOSES | 3 |
| response to elevated platelet cytosolic ca2+ | platelet | melanoma | platelet-derived growth factor | 1.49 | PRODUCES | 1 |
| tnf signaling pathway | tnf | tnfrsf4 | melanoma | 1.49 | ASSOCIATED_WITH | 1 |
| assembly of collagen fibrils and other multimeric structures | collagen | collagenase | melanoma | 1.48 | ASSOCIATED_WITH | 2 |
| tnf signaling pathway | tumor necrosis factor | tumor necrosis factor-alpha\|tnf | melanoma | 1.47 | AUGMENTS | 1 |
| tnf signaling pathway | tnf | tumor necrosis factor-alpha\|tnf | melanoma | 1.47 | AUGMENTS | 1 |
| interleukin-6 family signaling | interleukin-6 | interleukin-6 | melanoma cell | 1.46 | AFFECTS | 1 |
| tnf signaling pathway | tumor necrosis factor | melanoma | tumor necrosis factor ligand superfamily member 6\|faslg | 1.43 | PRODUCES | 1 |
| lung fibrosis | lung | melanoma | carcinoma, non-small-cell lung | 1.43 | AFFECTS | 1 |
| tnf signaling pathway | tumor necrosis factor | tumor necrosis factors | melanoma | 1.40 | PREDISPOSES | 1 |
| tnf signaling pathway | tumor necrosis factor | tumor necrosis factor-alpha\|tnf | melanoma | 1.40 | PART_OF | 1 |
| tnf signaling pathway | tnf | tumor necrosis factor-alpha\|tnf | melanoma | 1.40 | PART_OF | 1 |
| prostaglandin synthesis and regulation | prostaglandin | prostaglandins | melanoma | 1.37 | PREDISPOSES | 1 |
| interleukin-6 family signaling | interleukin-6 | interleukin-6 | melanoma | 1.37 | CAUSES | 1 |
| cytokine-cytokine receptor interaction | cytokine | cytokine | melanoma | 1.37 | PREVENTS | 1 |
| cytokine-cytokine receptor interaction | cytokine | cytokine | melanoma | 1.37 | PREVENTS | 1 |
| cytokine-cytokine receptor interaction | cytokine | cytokine | melanoma | 1.35 | NEG_AFFECTS | 1 |
| cytokine-cytokine receptor interaction | cytokine | cytokine | melanoma | 1.35 | NEG_AFFECTS | 1 |
| cytokine-cytokine receptor interaction | cytokine | cytokine | melanoma | 1.34 | AUGMENTS | 2 |
| cytokine-cytokine receptor interaction | cytokine | cytokine | melanoma | 1.34 | AUGMENTS | 2 |
| mir-509-3p alteration of yap1/ecm axis | extracellular matrix | extracellular matrix proteins | melanoma | 1.34 | ASSOCIATED_WITH | 2 |
| mir-509-3p alteration of yap1/ecm axis | extracellular matrix | extracellular matrix proteins | melanoma | 1.34 | ASSOCIATED_WITH | 2 |
| tnf signaling pathway | tnf | tnfrsf8 gene\|tnfrsf8 | melanoma | 1.32 | ASSOCIATED_WITH | 2 |
| cytokine-cytokine receptor interaction | cytokine | cytokine | melanoma cell | 1.31 | STIMULATES | 2 |
| cytokine-cytokine receptor interaction | cytokine | cytokine | melanoma cell | 1.31 | STIMULATES | 2 |
| tnf signaling pathway | tumor necrosis factor | tumor necrosis factor receptor 11b | melanoma | 1.31 | PREDISPOSES | 1 |
| apoptosis-related network due to altered notch3 in ovarian cancer | apoptosis | anti-apoptosis | melanoma | 1.28 | AFFECTS | 1 |
| assembly of collagen fibrils and other multimeric structures | collagen | melanoma cell | interstitial collagenase | 1.27 | PRODUCES | 1 |
| transcriptional misregulation in cancer | transcriptional | transcriptional regulation | melanoma | 1.25 | AFFECTS | 1 |
| tnf signaling pathway | tnf | tnfrsf10b gene\|tnfrsf10b | melanoma | 1.25 | PART_OF | 1 |
| prostaglandin synthesis and regulation | prostaglandin | melanoma cell | prostaglandins e | 1.25 | LOCATION_OF | 1 |
| tnf signaling pathway | tnf | melanoma cell | tnfrsf10b gene\|tnfrsf10b | 1.25 | LOCATION_OF | 1 |
| interleukin-6 family signaling | interleukin-6 | melanoma | interleukin-6\|il6 | 1.25 | PRODUCES | 1 |
| apoptosis-related network due to altered notch3 in ovarian cancer | apoptosis | casp8 and fadd-like apoptosis regulating protein | melanoma | 1.24 | ASSOCIATED_WITH | 1 |
| cytokine-cytokine receptor interaction | cytokine | melanoma | cytokine | 1.20 | NEG_PRODUCES | 1 |
| cytokine-cytokine receptor interaction | cytokine | melanoma | cytokine | 1.20 | NEG_PRODUCES | 1 |
| response to elevated platelet cytosolic ca2+ | platelet | melanoma cell | platelet-derived growth factor | 1.18 | PRODUCES | 1 |
| il-18 signaling pathway | interleukin-18 | melanoma cell | interleukin-18 | 1.16 | PRODUCES | 1 |
| assembly of collagen fibrils and other multimeric structures | collagen | procollagen | melanoma | 1.16 | ASSOCIATED_WITH | 1 |
| tnf signaling pathway | tnf | tnf protein, human\|tnf | melanoma cell | 1.15 | AUGMENTS | 1 |
| tnf signaling pathway | tumor necrosis factor | tumor necrosis factor ligand superfamily member 6\|faslg | melanoma | 1.14 | PART_OF | 1 |
| cytokine-cytokine receptor interaction | cytokine | cytokine | melanoma cell | 1.13 | PART_OF | 1 |
| cytokine-cytokine receptor interaction | cytokine | cytokine | melanoma cell | 1.13 | PART_OF | 1 |
| prostaglandin synthesis and regulation | prostaglandin | melanoma cell | prostaglandin-endoperoxide synthase 2 (prostaglandin g/h synthase and cyclooxygenase), human | 1.10 | LOCATION_OF | 1 |
| c-type lectin receptor signaling pathway | lectin | melanoma cell | galectin 3 | 1.08 | LOCATION_OF | 1 |
| assembly of collagen fibrils and other multimeric structures | collagen | melanoma cell | collagen type iv | 1.07 | LOCATION_OF | 1 |
| cytokine-cytokine receptor interaction | cytokine | cytokinesis of the fertilized ovum | melanoma | 1.06 | AFFECTS | 1 |
| cytokine-cytokine receptor interaction | cytokine | cytokinesis of the fertilized ovum | melanoma | 1.06 | AFFECTS | 1 |
| tnf signaling pathway | tnf | tnfrsf6b gene\|tnfrsf6b | melanoma | 1.06 | ASSOCIATED_WITH | 1 |
| cytokine-cytokine receptor interaction | cytokine | cytokine | melanoma cell | 1.05 | DISRUPTS | 1 |
| cytokine-cytokine receptor interaction | cytokine | cytokine | melanoma cell | 1.05 | DISRUPTS | 1 |
| micrornas in cancer | micrornas | micrornas | melanoma cell | 1.05 | DISRUPTS | 1 |
| assembly of collagen fibrils and other multimeric structures | collagen | collagen | melanoma cell | 1.05 | INTERACTS_WITH | 1 |
| response to elevated platelet cytosolic ca2+ | platelet | platelet activating factor | melanoma | 1.04 | AUGMENTS | 1 |
| apoptosis-related network due to altered notch3 in ovarian cancer | apoptosis | melanoma | apoptosis | 1.04 | MANIFESTATION_OF | 1 |
| cytokine-cytokine receptor interaction | cytokine | cytokine inducible sh2-containing protein\|cish | melanoma | 1.04 | ASSOCIATED_WITH | 1 |
| cytokine-cytokine receptor interaction | cytokine | cytokine inducible sh2-containing protein\|cish | melanoma | 1.04 | ASSOCIATED_WITH | 1 |
| tnf signaling pathway | tnf | melanoma cell | tnf gene\|tnf | 1.01 | LOCATION_OF | 1 |
| assembly of collagen fibrils and other multimeric structures | collagen | melanoma cell | collagen | 1.00 | INTERACTS_WITH | 1 |
| response to elevated platelet cytosolic ca2+ | platelet | melanoma | platelet aggregation | 1.00 | AFFECTS | 1 |
| tnf signaling pathway | tnf | tnf protein, human\|tnf | melanoma | 1.00 | DISRUPTS | 1 |
| transcriptional misregulation in cancer | transcriptional | transcriptional activation | melanoma | 0.00 | AFFECTS | 1 |
| transcriptional misregulation in cancer | transcriptional | melanoma cell | transcriptional activation | 0.00 | LOCATION_OF | 6 |
| senescence and autophagy in cancer | autophagy | melanoma | autophagy | 0.00 | AFFECTS | 2 |
| mir-509-3p alteration of yap1/ecm axis | extracellular matrix | extracellular matrix | melanoma | 0.00 | LOCATION_OF | 2 |
| il-18 signaling pathway | interleukin-18 | interleukin-18 | melanoma | 0.00 | ASSOCIATED_WITH | 2 |
| apoptosis-related network due to altered notch3 in ovarian cancer | apoptosis | melanoma | apoptosis | 0.00 | AFFECTS | 11 |
| cytokine-cytokine receptor interaction | cytokine | cytokine | melanoma | 0.00 | DISRUPTS | 1 |
| cytokine-cytokine receptor interaction | cytokine | cytokine | melanoma | 0.00 | PREDISPOSES | 1 |
| cytokine-cytokine receptor interaction | cytokine | cytokine | melanoma cell | 0.00 | AFFECTS | 1 |
| cytokine-cytokine receptor interaction | cytokine | cytokine | melanoma | 0.00 | AFFECTS | 1 |
| cytokine-cytokine receptor interaction | cytokine | cytokine | melanoma | 0.00 | ASSOCIATED_WITH | 32 |
| cytokine-cytokine receptor interaction | cytokine | melanoma | cytokine | 0.00 | PRODUCES | 7 |
| cytokine-cytokine receptor interaction | cytokine | melanoma cell | cytokine | 0.00 | LOCATION_OF | 3 |
| cytokine-cytokine receptor interaction | cytokine | melanoma cell | cytokine | 0.00 | PRODUCES | 13 |
| mir-509-3p alteration of yap1/ecm axis | extracellular matrix | extracellular matrix | melanoma | 0.00 | LOCATION_OF | 2 |
| assembly of collagen fibrils and other multimeric structures | collagen | collagen type iv | melanoma | 0.00 | ASSOCIATED_WITH | 1 |
| assembly of collagen fibrils and other multimeric structures | collagen | collagen | melanoma | 0.00 | ASSOCIATED_WITH | 8 |
| assembly of collagen fibrils and other multimeric structures | collagen | collagen type i | melanoma | 0.00 | ASSOCIATED_WITH | 1 |
| assembly of collagen fibrils and other multimeric structures | collagen | melanoma cell | collagen | 0.00 | LOCATION_OF | 1 |
| interleukin-6 family signaling | interleukin-6 | interleukin-6 | melanoma cell | 0.00 | STIMULATES | 1 |
| interleukin-6 family signaling | interleukin-6 | interleukin-6 | melanoma | 0.00 | ASSOCIATED_WITH | 13 |
| interleukin-6 family signaling | interleukin-6 | melanoma | interleukin-6 | 0.00 | PRODUCES | 3 |
| interleukin-6 family signaling | interleukin-6 | melanoma cell | interleukin-6 | 0.00 | LOCATION_OF | 4 |
| tnf signaling pathway | tumor necrosis factor | melanoma cell | tumor necrosis factors | 0.00 | LOCATION_OF | 1 |
| tnf signaling pathway | tumor necrosis factor | tumor necrosis factor receptor 11b | melanoma | 0.00 | ASSOCIATED_WITH | 1 |
| tnf signaling pathway | tumor necrosis factor | tumor necrosis factor-alpha\|tnf | melanoma | 0.00 | ASSOCIATED_WITH | 1 |
| tnf signaling pathway | tnf | tnfrsf5 gene\|cd40 | melanoma | 0.00 | ASSOCIATED_WITH | 2 |
| tnf signaling pathway | tnf | tumor necrosis factor-alpha\|tnf | melanoma | 0.00 | ASSOCIATED_WITH | 1 |
| tnf signaling pathway | tnf | tnf protein, human\|tnf | melanoma cell | 0.00 | STIMULATES | 1 |
| tnf signaling pathway | tnf | tnf protein, human\|tnf | melanoma | 0.00 | AUGMENTS | 1 |
| tnf signaling pathway | tnf | tnf protein, human\|tnf | melanoma | 0.00 | ASSOCIATED_WITH | 14 |
| tnf signaling pathway | tnf | tnf protein, human\|tnf | melanoma | 0.00 | PART_OF | 1 |
| tnf signaling pathway | tnf | tnf protein, human\|tnf | melanoma | 0.00 | CAUSES | 1 |
| tnf signaling pathway | tnf | tnf gene\|tnf | melanoma | 0.00 | ASSOCIATED_WITH | 3 |
| tnf signaling pathway | tnf | melanoma cell | tnf protein, human\|tnf | 0.00 | PRODUCES | 2 |
| tnf signaling pathway | tnf | melanoma cell | tnf protein, human\|tnf | 0.00 | LOCATION_OF | 6 |
| prostaglandin synthesis and regulation | prostaglandin | melanoma cell | prostaglandins | 0.00 | LOCATION_OF | 1 |
| prostaglandin synthesis and regulation | prostaglandin | melanoma | prostaglandins | 0.00 | PRODUCES | 1 |
| response to elevated platelet cytosolic ca2+ | platelet | platelet-derived growth factor receptor | melanoma | 0.00 | ASSOCIATED_WITH | 1 |
| response to elevated platelet cytosolic ca2+ | ca2+ | brca2 gene\|brca2 | melanoma | 0.00 | ASSOCIATED_WITH | 2 |
| response to elevated platelet cytosolic ca2+ | ca2+ | ca2 | melanoma cell | 0.00 | INTERACTS_WITH | 1 |
| response to elevated platelet cytosolic ca2+ | ca2+ | ca2 | melanoma | 0.00 | PART_OF | 1 |
| response to elevated platelet cytosolic ca2+ | ca2+ | ca2 | melanoma | 0.00 | ASSOCIATED_WITH | 1 |
| response to elevated platelet cytosolic ca2+ | ca2+ | melanoma cell | ca2 | 0.00 | LOCATION_OF | 1 |
| cytokine-cytokine receptor interaction | cytokine | cytokine | melanoma | 0.00 | DISRUPTS | 1 |
| cytokine-cytokine receptor interaction | cytokine | cytokine | melanoma | 0.00 | PREDISPOSES | 1 |
| cytokine-cytokine receptor interaction | cytokine | cytokine | melanoma cell | 0.00 | AFFECTS | 1 |
| cytokine-cytokine receptor interaction | cytokine | cytokine | melanoma | 0.00 | AFFECTS | 1 |
| cytokine-cytokine receptor interaction | cytokine | cytokine | melanoma | 0.00 | ASSOCIATED_WITH | 32 |
| cytokine-cytokine receptor interaction | cytokine | melanoma | cytokine | 0.00 | PRODUCES | 7 |
| cytokine-cytokine receptor interaction | cytokine | melanoma cell | cytokine | 0.00 | LOCATION_OF | 3 |
| cytokine-cytokine receptor interaction | cytokine | melanoma cell | cytokine | 0.00 | PRODUCES | 13 |
| lung fibrosis | lung | lung | melanoma | 0.00 | LOCATION_OF | 261 |
| lung fibrosis | lung | melanoma cell | lung | 0.00 | PART_OF | 3 |

**Supplementary Table 3. Validation of the 24 consensus pathways between PAGER, EnrichR results using BEERE.**

| **Term** | **Keywords** | **Subject** | **Object** | **Score** | **Predicate** | **S2O_CNT** |
| --- | --- | --- | --- | --- | --- | --- |
| viral protein interaction with cytokine and cytokine receptor | cytokine | cytokinesis of the fertilized ovum | melanoma | 19.76 | PROCESS_OF | 6 |
| nf-kappa b signaling pathway | nf-kappab | receptor activator of nf-kappab | melanoma cell | 10.63 | INTERACTS_WITH | 2 |
| viral protein interaction with cytokine and cytokine receptor | cytokine | recombinant cytokines | melanoma | 7.02 | NEG_AFFECTS | 1 |
| human papillomavirus infection | papillomavirus | melanoma cell | papillomavirus e7 proteins | 5.21 | PRODUCES | 1 |
| nf-kappa b signaling pathway | nf-kappab | receptor activator of nf-kappab | melanoma cell | 5.02 | STIMULATES | 1 |
| viral protein interaction with cytokine and cytokine receptor | cytokine | melanoma cell | cytokinesis of the fertilized ovum | 4.81 | LOCATION_OF | 4 |
| gastrin signaling pathway | gastrin | tetragastrin\|cck | melanoma | 4.45 | ASSOCIATED_WITH | 1 |
| selenium micronutrient network | selenium | selenium | melanoma | 4.36 | PREDISPOSES | 3 |
| viral protein interaction with cytokine and cytokine receptor | cytokine | cytokine gene | melanoma cell | 4.28 | ADMINISTERED_TO | 1 |
| nf-kappa b signaling pathway | nf-kappab | nf-kappab-inducing kinase | melanoma cell | 4.15 | STIMULATES | 1 |
| nf-kappa b signaling pathway | nf-kappab | nf-kappab-inducing kinase | melanoma cell | 3.97 | INTERACTS_WITH | 1 |
| viral protein interaction with cytokine and cytokine receptor | cytokine | cytokine inducible sh2-containing protein\|cish | melanoma | 3.80 | PREDISPOSES | 1 |
| lipid and atherosclerosis | lipid | melanoma cell | glycosphingolipids | 3.53 | LOCATION_OF | 2 |
| viral protein interaction with cytokine and cytokine receptor | cytokine | melanoma cell | cytokine inducible sh2-containing protein\|cish | 3.49 | PRODUCES | 1 |
| human cytomegalovirus infection | cytomegalovirus | cytomegalovirus dna | melanoma | 3.30 | NEG_ASSOCIATED_WITH | 1 |
| viral protein interaction with cytokine and cytokine receptor | cytokine | melanoma cell | cytokine | 3.30 | INTERACTS_WITH | 4 |
| viral protein interaction with cytokine and cytokine receptor | viral protein | melanoma cell | viral proteins | 3.13 | NEG_PRODUCES | 1 |
| viral protein interaction with cytokine and cytokine receptor | cytokine | melanoma cell | cytokine gene | 3.02 | LOCATION_OF | 2 |
| viral protein interaction with cytokine and cytokine receptor | cytokine | cytokine | melanoma | 3.02 | PART_OF | 5 |
| lipid and atherosclerosis | lipid | lipid raft | melanoma cell | 2.99 | PART_OF | 1 |
| viral protein interaction with cytokine and cytokine receptor | cytokine | melanoma cell | cytokine gene | 2.94 | INTERACTS_WITH | 1 |
| viral protein interaction with cytokine and cytokine receptor | cytokine | melanoma cell | small inducible cytokine a21\|ccl21 | 2.87 | PRODUCES | 1 |
| g alpha (i) signaling events | g protein alpha | gtp-binding protein alpha subunits | melanoma | 2.57 | ASSOCIATED_WITH | 1 |
| viral protein interaction with cytokine and cytokine receptor | cytokine | cytokine | melanoma cell | 2.55 | INTERACTS_WITH | 5 |
| lipid and atherosclerosis | lipid | glycolipids | melanoma cell | 2.41 | PART_OF | 1 |
| glucocorticoid receptor pathway | glucocorticoid | glucocorticoid receptor | melanoma | 2.41 | ASSOCIATED_WITH | 5 |
| viral protein interaction with cytokine and cytokine receptor | cytokine | cytokine | melanoma cell | 2.34 | NEG_AFFECTS | 1 |
| glucocorticoid receptor pathway | glucocorticoid | glucocorticoid receptor | melanoma | 2.33 | PART_OF | 2 |
| lipid and atherosclerosis | lipid | melanoma cell | glycolipids | 2.22 | PRODUCES | 1 |
| selenium micronutrient network | selenium | selenium | melanoma cell | 2.18 | DISRUPTS | 1 |
| viral protein interaction with cytokine and cytokine receptor | viral protein | melanoma cell | viral proteins | 2.17 | INTERACTS_WITH | 1 |
| lipid and atherosclerosis | lipid | sulfoglycosphingolipids | melanoma | 2.16 | ASSOCIATED_WITH | 2 |
| viral protein interaction with cytokine and cytokine receptor | cytokine | melanoma cell | cytokine inducible sh2-containing protein\|cish | 1.89 | LOCATION_OF | 1 |
| selenium micronutrient network | selenium | selenium | melanoma | 1.87 | DISRUPTS | 1 |
| viral protein interaction with cytokine and cytokine receptor | cytokine | cytokine gene | melanoma | 1.75 | ASSOCIATED_WITH | 3 |
| viral protein interaction with cytokine and cytokine receptor | viral protein | viral proteins | melanoma | 1.65 | ASSOCIATED_WITH | 2 |
| glucocorticoid receptor pathway | glucocorticoid | melanoma cell | glucocorticoids | 1.62 | LOCATION_OF | 2 |
| viral protein interaction with cytokine and cytokine receptor | cytokine | cytokine | melanoma | 1.55 | TREATS | 1 |
| lipid and atherosclerosis | lipid | melanoma cell | sphingolipids | 1.38 | LOCATION_OF | 1 |
| viral protein interaction with cytokine and cytokine receptor | cytokine | cytokine | melanoma | 1.37 | PREVENTS | 1 |
| viral protein interaction with cytokine and cytokine receptor | cytokine | cytokine | melanoma | 1.35 | NEG_AFFECTS | 1 |
| viral protein interaction with cytokine and cytokine receptor | cytokine | cytokine | melanoma | 1.34 | AUGMENTS | 2 |
| lipid and atherosclerosis | lipid | sphingolipids | melanoma | 1.32 | ASSOCIATED_WITH | 2 |
| viral protein interaction with cytokine and cytokine receptor | cytokine | cytokine | melanoma cell | 1.31 | STIMULATES | 2 |
| selenium micronutrient network | selenium | selenium | melanoma | 1.30 | PREVENTS | 1 |
| glucocorticoid receptor pathway | glucocorticoid | glucocorticoid receptor | melanoma | 1.30 | AFFECTS | 1 |
| glucocorticoid receptor pathway | glucocorticoid | glucocorticoids | melanoma cell | 1.27 | AFFECTS | 1 |
| gastrin signaling pathway | gastrin | gastrin releasing peptide | melanoma | 1.27 | ASSOCIATED_WITH | 1 |
| viral protein interaction with cytokine and cytokine receptor | cytokine | melanoma | cytokine | 1.20 | NEG_PRODUCES | 1 |
| lipid and atherosclerosis | lipid | melanoma cell | glycolipids | 1.16 | LOCATION_OF | 1 |
| viral protein interaction with cytokine and cytokine receptor | cytokine | cytokine | melanoma cell | 1.13 | PART_OF | 1 |
| lipid and atherosclerosis | lipid | glycosphingolipids | melanoma | 1.09 | ASSOCIATED_WITH | 1 |
| glucocorticoid receptor pathway | glucocorticoid | glucocorticoids | melanoma | 1.07 | NEG_TREATS | 1 |
| viral protein interaction with cytokine and cytokine receptor | cytokine | cytokinesis of the fertilized ovum | melanoma | 1.06 | AFFECTS | 1 |
| selenium micronutrient network | selenium | selenium | melanoma | 1.06 | AFFECTS | 1 |
| viral protein interaction with cytokine and cytokine receptor | cytokine | cytokine | melanoma cell | 1.05 | DISRUPTS | 1 |
| viral protein interaction with cytokine and cytokine receptor | cytokine | cytokine inducible sh2-containing protein\|cish | melanoma | 1.04 | ASSOCIATED_WITH | 1 |
| lipid and atherosclerosis | lipid | melanoma cell | atpase, aminophospholipid transporter-like, class i, type 8a, member 2\|atp8a2 | 1.02 | INTERACTS_WITH | 1 |
| lipid and atherosclerosis | lipid | glycolipids | melanoma | 1.00 | ASSOCIATED_WITH | 1 |
| viral protein interaction with cytokine and cytokine receptor | cytokine | melanoma | cytokine | 0.00 | PRODUCES | 7 |
| viral protein interaction with cytokine and cytokine receptor | cytokine | melanoma cell | cytokine | 0.00 | PRODUCES | 13 |
| viral protein interaction with cytokine and cytokine receptor | cytokine | melanoma cell | cytokine | 0.00 | LOCATION_OF | 3 |
| viral protein interaction with cytokine and cytokine receptor | cytokine | cytokine | melanoma cell | 0.00 | AFFECTS | 1 |
| viral protein interaction with cytokine and cytokine receptor | cytokine | cytokine | melanoma | 0.00 | AFFECTS | 1 |
| viral protein interaction with cytokine and cytokine receptor | cytokine | cytokine | melanoma | 0.00 | ASSOCIATED_WITH | 32 |
| viral protein interaction with cytokine and cytokine receptor | cytokine | cytokine | melanoma | 0.00 | DISRUPTS | 1 |
| viral protein interaction with cytokine and cytokine receptor | cytokine | cytokine | melanoma | 0.00 | PREDISPOSES | 1 |
| glucocorticoid receptor pathway | glucocorticoid | glucocorticoids | melanoma | 0.00 | TREATS | 1 |
| glucocorticoid receptor pathway | glucocorticoid | glucocorticoids | melanoma | 0.00 | ASSOCIATED_WITH | 4 |
| selenium micronutrient network | selenium | selenium | melanoma | 0.00 | ASSOCIATED_WITH | 2 |
| lipid and atherosclerosis | lipid | lipids | melanoma cell | 0.00 | PART_OF | 1 |
| lipid and atherosclerosis | lipid | lipids | melanoma | 0.00 | ASSOCIATED_WITH | 5 |
| lipid and atherosclerosis | lipid | lipid raft | melanoma | 0.00 | LOCATION_OF | 1 |
| lipid and atherosclerosis | lipid | melanoma cell | atpase, aminophospholipid transporter-like, class i, type 8a, member 2\|atp8a2 | 0.00 | LOCATION_OF | 1 |

**Supplementary Table 4. Validation of the consensus pathways between PAGER and WebGestaltR results using BEERE.**

| **Term** | **Keywords** | **Subject** | **Object** | **Score** | **Predicate** | **S2O_CNT** |
| --- | --- | --- | --- | --- | --- | --- |
| interleukin-10 signaling | interleukin-10 | melanoma cell | interleukin-10\|il10 | 4.74 | PRODUCES | 3 |
| interleukin-4 and interleukin-13 signaling | interleukin-4 | recombinant interleukin-4\|il4 | melanoma | 4.12 | TREATS | 1 |
| interleukin-10 signaling | interleukin-10 | interleukin-10\|il10 | melanoma | 3.68 | AUGMENTS | 1 |
| interleukin-4 and interleukin-13 signaling | interleukin-4 | interleukin-4\|il4 | melanoma cell | 3.64 | DISRUPTS | 1 |
| interleukin-4 and interleukin-13 signaling | interleukin-4 | recombinant interleukin-4 | melanoma | 3.62 | ASSOCIATED_WITH | 1 |
| interleukin-4 and interleukin-13 signaling | interleukin-13 | interleukin-13 | melanoma | 3.56 | NEG_ASSOCIATED_WITH | 1 |
| interleukin-10 signaling | interleukin-10 | interleukin-10\|il10 | melanoma cell | 3.30 | INTERACTS_WITH | 1 |
| interleukin-4 and interleukin-13 signaling | interleukin-4 | interleukin-4 | melanoma | 2.68 | PART_OF | 2 |
| interleukin-4 and interleukin-13 signaling | interleukin-4 | recombinant interleukin-4 | melanoma | 2.64 | TREATS | 1 |
| interleukin-4 and interleukin-13 signaling | interleukin-4 | interleukin-4 | melanoma cell | 2.24 | AFFECTS | 1 |
| interleukin-4 and interleukin-13 signaling | interleukin-4 | interleukin-4 | melanoma cell | 2.24 | PART_OF | 1 |
| interleukin-10 signaling | interleukin-10 | interleukin-10 | melanoma | 2.15 | PREDISPOSES | 2 |
| interleukin-10 signaling | interleukin-10 | interleukin-10\|il10 | melanoma | 2.14 | ASSOCIATED_WITH | 3 |
| interleukin-10 signaling | interleukin-10 | interleukin-10 | melanoma | 1.85 | NEG_ASSOCIATED_WITH | 1 |
| interleukin-10 signaling | interleukin-10 | melanoma cell | interleukin-10 | 1.83 | LOCATION_OF | 4 |
| interleukin-4 and interleukin-13 signaling | interleukin-4 | interleukin-4 | melanoma | 1.74 | NEG_ASSOCIATED_WITH | 1 |
| interleukin-10 signaling | interleukin-10 | melanoma | interleukin-10\|il10 | 1.62 | PRODUCES | 1 |
| interleukin-4 and interleukin-13 signaling | interleukin-4 | melanoma cell | interleukin-4\|il4 | 1.24 | PRODUCES | 1 |
| interleukin-4 and interleukin-13 signaling | interleukin-4 | interleukin-4 | melanoma | 1.12 | AFFECTS | 1 |
| interleukin-4 and interleukin-13 signaling | interleukin-4 | melanoma | interleukin-4 | 0.00 | PRODUCES | 3 |
| interleukin-4 and interleukin-13 signaling | interleukin-4 | melanoma cell | interleukin-4 | 0.00 | PRODUCES | 3 |
| interleukin-10 signaling | interleukin-10 | interleukin-10 | melanoma | 0.00 | ASSOCIATED_WITH | 7 |
| interleukin-10 signaling | interleukin-10 | melanoma | interleukin-10 | 0.00 | PRODUCES | 2 |
| interleukin-10 signaling | interleukin-10 | melanoma cell | interleukin-10 | 0.00 | PRODUCES | 4 |

Supplementary Table 5. The 6 consensus pathways among EnrichR, and WebGestaltR results with PubMed literature support. W vs. P represents the term similarities between WebGestaltR and PAGER results. P vs. E represents the term similarities between PAGER and EnrichR results. W vs. E represents the term similarities between WebGestaltR and EnrichR. $\boldsymbol{k}$ represents the citations of “melanoma” and the keywords from a pathway. $\boldsymbol{OR}$ represents the odds ratio. Score represents the $\boldsymbol{PubMed score}$. PMID represents one PubMed ID example from each entry.

| **Term** | **W vs. E** | **Keywords** | **k** | **OR** | **Score** |
| --- | --- | --- | --- | --- | --- |
| iron metabolism in placenta | 100% | iron metabolism in placenta | 1 | 0.047 | 6.87E-10 |
| platelet degranulation | 100% | platelet degranulation | 8 | 0.193 | 7.79E-11 |
| extracellular matrix organization | 100% | extracellular matrix organization | 62 | 0.270 | 3.34E-39 |
| synthesis of prostaglandins (pg) and thromboxanes (tx) | 100% | prostaglandins, thromboxanes | 11 | 0.041 | 5.18E-99 |
| differentiation pathway | 100% | differentiation pathway | 771 | 0.414 | 2.97E-175 |
| collagen formation | 100% | collagen formation | 1007 | 0.245 | 0.00E+00 |
